# Cross-talk between signaling pathways: The link between plant secondary metabolite production and wounding stress response

**DOI:** 10.1038/srep08608

**Published:** 2015-02-25

**Authors:** Daniel A. Jacobo-Velázquez, Mauricio González-Agüero, Luis Cisneros-Zevallos

**Affiliations:** 1 Department of Horticultural Sciences, Texas A‌&‌M University, Vegetable ‌&‌ Fruit Improvement Center, College Station, TX 77843-2133, U.S.A; 2 Institute of Agricultural Research (INIA-CRI La Platina), Santiago, Chile

## Abstract

Plants subjected to wounding stress produce secondary metabolites. Several of these metabolites prevent chronic diseases and can be used as colorants, flavors, and as antimicrobials. This wound-induced production of plant secondary metabolites is mediated by signaling-molecules such as reactive oxygen species (ROS), ethylene (ET) and jasmonic acid (JA). However, their specific role and interactions that modulate the wound-respond in plants is not fully understood. In the present study, a subtractive cDNA library was generated, to better understand the global response of plants to wounding stress. Carrot (*Daucus carota*) was used as a model system for this study. A total of 335 unique expressed sequence tags (ESTs) sequences were obtained. ESTs sequences with a putative identity showed involvement in stress-signaling pathways as well as on the primary and secondary metabolism. Inhibitors of ROS biosynthesis, ET action, and JA biosynthesis alone and in combination were applied to wounded-carrots in order to determine, based on relative gene expression data, the regulatory role of ET, JA, and ROS on the wound-response in plants. Our results demonstrate that ROS play a key role as signaling-molecules for the wound-induced activation of the primary and secondary metabolism whereas ET and JA are essential to modulate ROS levels.

Plants are continuously exposed to mechanical wounding and thus they have a well-designed metabolic machinery that rapidly respond synthesizing proteins and secondary metabolites that hamper pathogens invasion at the injured site. Several plant secondary metabolites have potential application on the treatment of different degenerative diseases. Numerous crop plants such as potato^1^, rice^2^, and tomato^3^, have been genetically engineered to produce higher levels of health-promoting phytochemicals. However, metabolic engineering is technically complex and thus additional and more practical approaches to produce plant bioactives are needed.

The wound-induced production of secondary metabolites can be exploited as a practical and effective strategy to produce high levels of bioactive compounds^4^,^5^. For instance, carrots respond to wounding stress producing and accumulating caffeoylquinic acids^5^,^6^, which are phenolic compounds (PC) with potential applications in the treatment and prevention of chronic-diseases such as obesity^7^, diabetes^8^, hepatitis B^9^, cardiovascular diseases^10^, neurodegenerative diseases^11^, and HIV^12^,^13^.

Little is known on the molecular mechanism governing the wound-induced production and accumulation of PC in plants. This stress response seems to be the result of the activation of the phenylpropanoid metabolism together with those metabolic pathways involved in the supplementation of carbons skeletons needed for phenolics biosynthesis such as the glycolysis, the oxidative pentose phosphate pathway (OPPP), and the shikimate pathway. In a previous report, the physiological role of ROS on the wound-induced accumulation of phenolics in carrots was discussed^5^. In addition to ROS other signaling molecules such as ET and JA have been reported to mediate stress responses in plants^14^,^15^,^16^. However, their cross-talk and interactions that modulate the wound-response remains unknown.

In the present study, a subtractive cDNA library was generated to better understand the global response of carrots to wounding stress. The relative expression through time of genes identified with putative function related with the biosynthesis of ROS, ET, and JA as well as those related with the primary and secondary metabolism was evaluated and the promptness of their response to wounding stress was determined. In addition, inhibitors of ROS biosynthesis (diphenyleneiodonium chloride, DPI), ET action (1-methylcyclopropene, 1-MCP), and JA biosynthesis (phenidone, PHEN) alone and in combination were applied to wounded-carrot tissue in order to determine, based on relative gene expression data, the regulatory role of ET, JA, and ROS on the wound-response in plants. The methodological approach used in the present study allows the generation of information that would take years by applying conventional biotechnology methods of gene suppressions, activation or silencing.

In the present research we addressed the following questions: (i) how ROS, ET, and JA interact to mediate the wound-response in plants?; (ii) how ROS, ET, and JA modulate genes with putative function related with the primary and secondary metabolism in wounded-plant tissue?; (iii) how ROS, ET, and JA affect the wound-induced production of phenolic compounds in carrots?. The information generated in this research can be useful to envisage strategies to enhance the concentration of valuable bioactive compounds in plants. Enhancing crop value through wounding stress will be attractive to the fresh and processed functional foods, dietary supplements, cosmetics and agrochemical markets, and will have a positive impact on the dietary intake of antioxidants.

## Results and Discussion

### Subtractive cDNA library construction

To increase our understanding on the molecular mechanisms involved in the wound-response in plants a subtractive cDNA library was constructed. Wounded-carrot tissue was stored at 20°C for 24 h. To obtain the subtractive cDNA library, the suppression subtractive hybridization (SSH) technique was performed with control samples (non-wounded, time 0 h samples) as the driver and wounded-samples as the tester. A total of 1,056 clones from the forward library were selected randomly and after sequencing and BLAST searching 335 unique expressed sequence tags (ESTs) sequences were obtained. ESTs were classified according to their metabolic function (fig. S1). The wound induced activation of genes involved in the biosynthesis of stress-signaling molecules, primary and secondary metabolism was confirmed by real-time quantitative reverse transcription PCR (qRT-PCR) (tables S1–S4). The whole EST forward library is shown as supplementary information (table S5).

### A complex cross-talk between ROS, ET, and JA signaling pathways modulate the wound-response in carrots

It is known that ROS, ET, and JA are important signal mediators of the wound-response in plants. Previous investigations have demonstrated that ROS and JA induce the activation of 1-aminocyclopropane-1-carboxylate (ACC) synthase in wounded-winter squash^15^. Likewise, it has been proposed that hydrogen peroxide (H_2_O_2_) acts as a second messenger for the wound-induced activation of defense genes in tomato plants^16^. However, it is not well understood if the production of ET, JA, and ROS occurs simultaneously or if their wound-induced production is dependent or regulated by each other.

Based on gene expression data, few minutes after wounding take place, manganese superoxide dismutase (MnSOD), glycolate oxidase (GOX), and ACC synthase genes are activated (figs. S2–S3), suggesting that ROS and ET are rapidly produced after wounding. Similar observations on the wound-induced production of ROS and ET have been reported for *Arabidopsis* leaves^17^, winter squash^18^, tomato^19^, and carrots^5^. Compared with genes related with the biosynthesis of ROS and ET, which showed activation during the first hours of storage, JA biosynthesis genes showed later activation, suggesting that JA is produced after ROS and ET (fig. S4). These results are in accordance with a previous report on LOX activity, ROS, and ET production in sliced carrots stored at 15°C^20^. The author reported that during the first 4 h of storage ROS and ET are produced as an immediate response to wounding, whereas a significant increase in LOX activity (involved on JA production) is observed at 12 h of storage.

**Figure 1:**
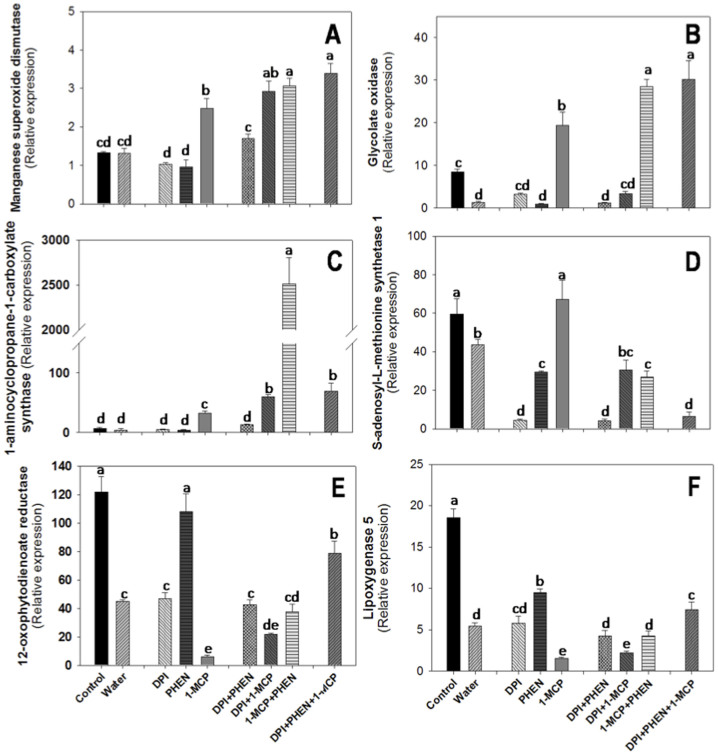
Effect of diphenyleneiodonium chloride (DPI), phenidone (PHEN) and 1-methylcyclopropene (1-MCP) on the relative expression of genes with putative function related with the biosynthesis of stress signaling molecules. Manganese superoxide dismutase (MnSOD, A); Glycolate oxidase (GOX, B); 1-aminocyclopropane-1-carboxylate synthase (ACC synthase, C); S-adenosyl-methionine synthetase 1 (SAM synthetase, D); 12-oxophytodienoate reductase (12-OPDA reductase, E); lipoxygenase 5 (LOX5, E). MnSOD, GOX and ACC synthase relative expression is shown at 30 min after wounding. 12-OPDA reductase relative expression was collected at 6 h after wounding. SAM synthetase and LOX 5 relative expression was determined at 12 h after wounding. Data represents the mean of 4 replicates ± standard error of the mean. Bars with different letters indicate statistical difference by the LSD test (p < 0.05).

In order to elucidate the role of ROS, ET, and JA as well as a potential cross-talk between them on the regulation of the wound-response in plants, experiments using DPI, PHEN, and 1-MCP as ROS, ET, and JA inhibitors, respectively, were conducted. The effect of these inhibitors on the relative expression of MnSOD, GOX, ACC synthase, S-adenosyl-L-methionine (SAM) synthetase, lipoxygenase 5 (LOX5), and 12-oxophytodienoate (12-OPDA) reductase genes was studied (Fig. 1). According to our results of the previous experimental stage (figs. S2–S4), these genes seem to have a primordial role on the production and regulation of ROS, ET, and JA in carrots. The relative expression of each gene is presented at a particular time after wounding based on their specific response time after wounding (figs. S2–S4). Shredded-carrot tissue was submerged on a solution containing either DPI or PHEN. Combinatorial treatments using more than one inhibitor at a time were also conducted in order to elucidate cross-talks between ROS, ET, and JA. Shredded-carrots exposed to air were used as control samples for 1-MCP treatments. Shredded-carrots washed with water were used as control sample for samples washed on DPI or PHEN solutions either alone or in combination with one another or treated with 1-MCP. We decided to use 2 different control samples because it has been reported that upon the application of wounding the cytosolic content of plant cells, containing molecules such as adenosine triphosphate (ATP) that may trigger the wound-response in plants, is liberated to the tissue matrix^5^,^21^. Therefore, the washing of such signals may have a negative effect on the wound-induced activation of some genes related with the wound-response.

The use of DPI and PHEN individually did not generate a significant effect on the relative expression of MnSOD and GOX genes (Fig. 1). However, the use of 1-MCP resulted in a significant increase in the expression of both genes, indicating a negative regulation of ET over MnSOD and GOX expression (Fig. 1). Moreover, in all combinational treatments containing 1-MCP an increase on the expression of MnSOD was observed, further confirming a negative regulation of ET over the expression of the gene. Similar observations have been reported for broccoli florets where the application of 1-MCP increased the activity of SOD enzyme^22^. This data indicate that while ET and ROS production occurs simultaneously as an immediate response to wounding^20^, ET plays an important role on modulating ROS levels by controlling MnSOD activity and gene expression^22^ as an early and late response. In the case of GOX putative gene, when 1-MCP was used in combination with PHEN, the wound-induced activation of the gene was even higher (Fig. 1B), reflecting a cross-talk between ET and JA that negatively regulate ROS production, probably through a GOX transcriptional repressor. These results indicate that ET has at least two different ways to modulate the levels of ROS in plants, by itself regulating MnSOD and GOX, and in conjunction with JA regulating GOX. To our knowledge, these findings have not been previously reported.

Regarding the putative ACC synthase gene, the use of 1-MCP generated a significant increase in its relative expression (Fig. 1C). Since ACC synthase gene is related with the production of ET, a negative feedback regulation of ET is occurring. Similar observations have been reported for ‘Saijo' persimmon fruit where the application of 1-MCP produced an increase in the expression of *DK-ACS2* gene as well as on ACC synthase activity^23^. When 1-MCP was used in combination with PHEN, the increase on relative expression was much higher, indicating as in the case of GOX gene, a synergistic effect of ET and JA that decreases the expression of ACC synthase. Previous reports indicate that the presence of ET and JA favor the expression of transcriptional repressors such as certain ethylene-responsive transcription factors (ERFs)^24^. These ERFs may be the cause of the decreased expression of ACC synthase observed. However, the presence of ROS is necessary to observe this effect. When DPI was used together with PHEN and 1-MCP the effect on ACC synthase relative expression induced by wounding was much lower, indicating a complex cross-talk between ROS, ET, and JA that regulates the wound-induced gene expression of ACC synthase on the wounded-tissue.

Concerning SAM synthetase 1 putative gene (Fig. 1D), the use of DPI and PHEN either individually or in combination inhibited its wound-induced expression, indicating that the presence of ROS and JA on the tissue favors the production of ET at late stages of the wounding stress response. On the other hand, the use of 1-MCP had no significant effect on the expression. Although both genes (ACC synthase and SAM synthetase) have putative function related with the production of ET, it seems that their regulation is related to separate mechanisms. While the wound-induced activation of ACC synthase (an early-response gene) seems to be independent of ROS and JA, these two signaling molecules up-regulate the wound-induced expression of SAM synthetase 12 h after wounding.

The application of DPI had no significant effect on the expression of 12-OPDA (Fig. 1E) and LOX (Fig. 1F) genes as compared with water controls. Likewise, the use of PHEN generated a significant increase on the wound-induced activation of both genes as compared with water controls. Since 12-OPDA reductase and LOX are directly related with the production of JA, the presence of such signaling molecule may generate feedback regulation. On the other hand, the use of 1-MCP produced a significant decrease on the relative expression of 12-OPDA gene (Fig. 1E), suggesting that ET production favors the stress-induced biosynthesis of JA. Compared with the 1-MCP+PHEN treatment, an increase in the relative expression of 12-OPDA reductase and LOX 5 genes was observed when the three signaling molecules were blocked; implying that in the absence of ET and JA, the absence of ROS favors the wound-induced activation of both genes.

**Figure 2:**
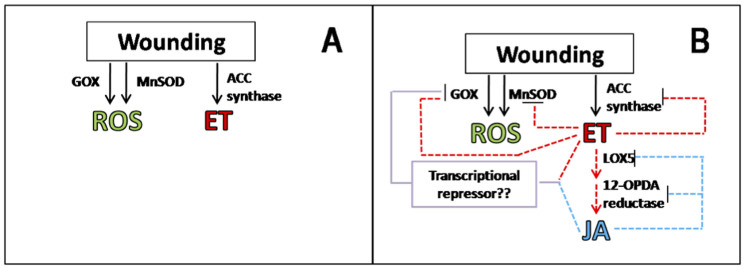
Hypothetical model explaining the cross-talk interaction of reactive oxygen species (ROS), ethylene (ET) and jasmonic acid (JA) on the regulation of the wound-response in carrot. Early response (A) and late response (B).

Based on the relative expression data previously presented, a theoretical model explaining a complex cross-talk between ROS, ET and JA that regulates the wounding stress responses in plants is proposed (Fig. 2). It is known that after wounding take place, ROS and ET production occurs simultaneously^5^,^21^, as an immediate wound-response, likely by immediate activation of ROS and ET producing enzymes. The model proposed herein suggests that as the concentration of ROS and ET in the tissue increases (early response, Fig. 2A), their role as signaling molecules through a complex cross-talk becomes evident (Fig. 2B). The synthesis of JA via LOX 5 and 12-OPDA reductase is favored due to the accumulation of ET. Additionally, ET can down-regulate ACC synthase, likely with a feedback mechanism, preventing an excessive accumulation of ET in the tissue. As JA accumulates on the tissue several repression mechanisms take place in order to regulate the concentration of ROS, ET, and JA (Fig. 2B). ET in combination with JA may down-regulate the expression of GOX, probably via a transcriptional repressor. Similarly, ET and JA can modulate its own concentration down-regulating ACC synthase, LOX 5, and 12-OPDA reductase, likely with a negative feedback mechanism (Fig. 1).

The developed model indicates that, as expected, the role of ROS, ET, and JA on the regulation of the wounding stress responses involves a complex cross-talk among these signaling molecules. Although some studies reported in the literature overlook the role of ET as signaling molecule, the present study demonstrated that ET actually has a major role on the regulation of ROS and JA levels. Similarly, JA has a major role in conjunction with ET, preventing the excessive accumulation of ROS. As discussed before^5^, high levels of ROS, particularly H_2_O_2_ may result toxic and even lethal to the cell^25^. Therefore, the regulatory mechanism exerted by JA and ET may act in combination with the ascorbate peroxidase and catalase detoxifying activity to prevent the excessive accumulation of H_2_O_2_. To our knowledge, no previous research work has reported cross-talks between ROS, ET and JA that regulates the wounding response on plants.

### ROS play a major role on the wound-induced activation of primary and secondary metabolism related genes in carrots

The relative expression of genes with putative function identified as 3-deoxy-D-arabino-heptulosanate synthase (DAHP synthase) and 5-enolpyrovylshikimate 3-phosphate synthase (EPSP synthase), which are related with the primary metabolism, increased progressively through time by the effect of wounding (fig. S5). Similarly, the relative expression of genes with putative function related with the secondary metabolism showed a progressive increase through time (fig. S6).

**Figure 3:**
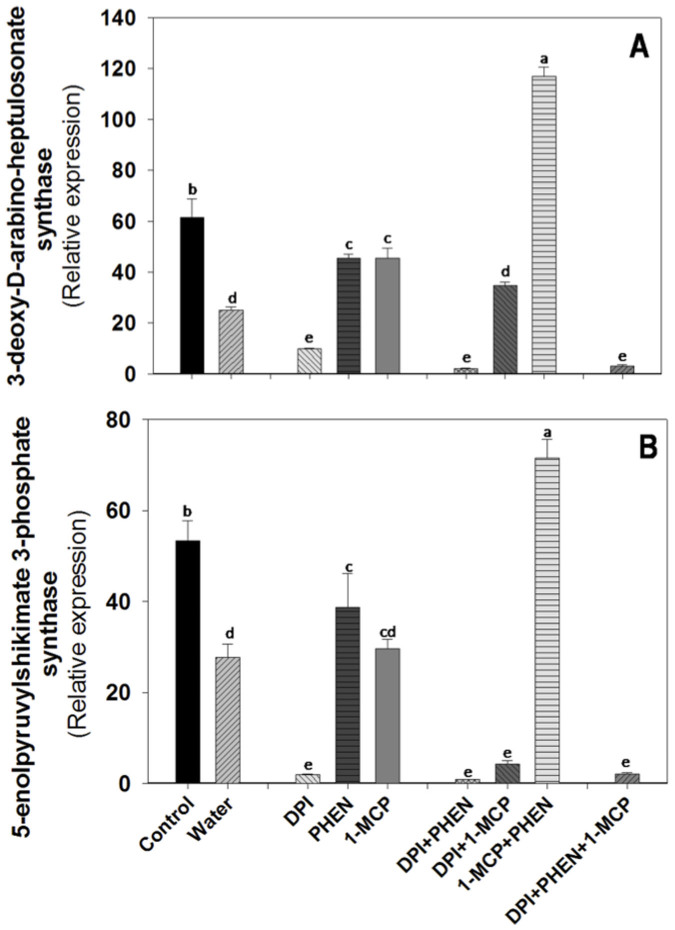
Effect of diphenyleneiodonium chloride (DPI), phenidone (PHEN) and 1-methylcyclopropene (1-MCP) on the relative expression of genes with putative function related with the primary metabolism in wounded carrot tissue. 3-deoxy-D-arabino-heptulosanate synthase (DAHP synthase, A); 5-enolpyrovylshikimate 3-phosphate synthase (EPSP synthase, B). Relative expression was obtained 24 h after wounding. Data represents the mean of 4 replicates ± standard error of the mean. Bars with different letters indicate statistical difference by the LSD test (p < 0.05).

**Figure 4:**
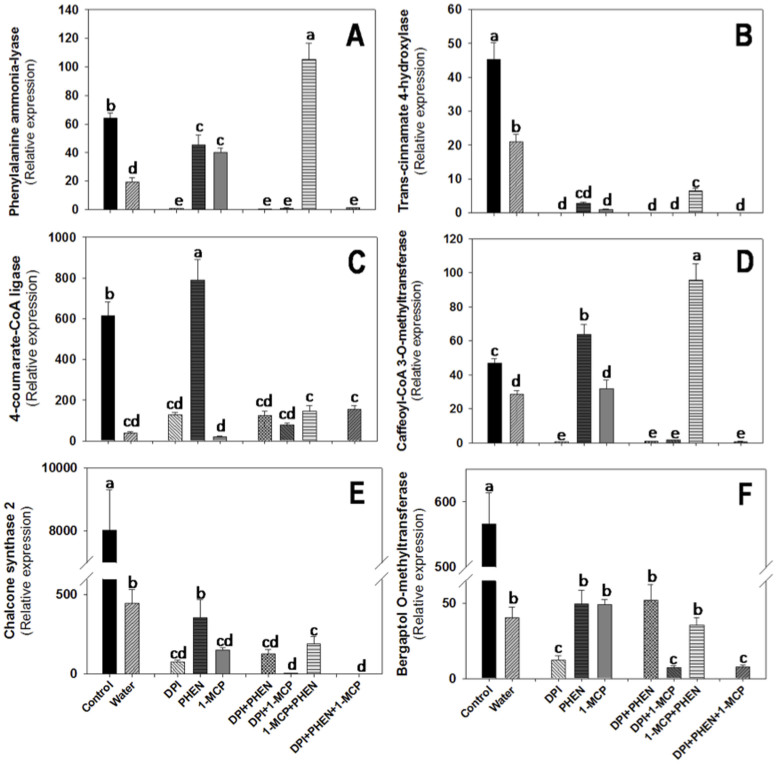
Effect of diphenyleneiodonium chloride (DPI), phenidone (PHEN) and 1-methylcyclopropene (1-MCP) on the relative expression of genes with putative function related with the secondary metabolism in wounded-carrot tissue. Phenylalanine ammonia-lyase (PAL, A); *trans*-cinnamate 4-hydroxylase (C4H, B); 4-coumarate-CoA ligase (4CL, C); caffeoyl-CoA 3-O-methyltransferase (CCoAOMT, D); chalcone synthase 2 (CHS, E); bergaptol O-methyltransferase (BOMT, F). Relative expression was obtained 24 h after wounding. Data represents the mean of 4 replicates ± standard error of the mean. Bars with different letters indicate statistical difference by the LSD test (p < 0.05).

As mention earlier, the activation of several defense genes in plants subjected to abiotic stresses is in many instances determined by regulatory mechanisms related to ET and JA signaling pathways^14^,^26^. Additionally, it has been demonstrated that ROS play an important role on the modulation of secondary metabolites biosynthesis^5^. In order to elucidate potential interactions between ROS, ET, and JA for the modulation of genes related to the primary and secondary metabolism, the expression of these genes was evaluated on shredded-carrots treated with DPI, PHEN or 1-MCP, either alone or in combination (Figs. 3–4). As observed in Fig. 1, treating the tissue with the inhibitors produced alterations in the relative expression of genes with putative function related with the biosynthesis of ROS, ET, and JA. The use of individual inhibitors and their combinations would result in an alteration of the concentration of signals according to cross-talk describe earlier. Those effects of the resultant signals concentrations exerted by the application of the inhibitors were considered in the discussion of the following sections. The relative expression of primary and secondary metabolites related genes was determined 24 h after wounding; time selected based on the results previously presented (figs. S5–S6).

Samples treated with DPI showed a significant decrement in the wound-induced activation of DAHP synthase and EPSP synthase genes, with putative function related with the primary metabolism, indicating that ROS play a major role on the wound-induced activation of the shikimic acid pathway (Fig. 3). Likewise, a decrease in expression was observed when shredded-carrots were treated with 1-MCP, indicating that ET also up-regulates these genes. However, when 1-MCP was used in combination with PHEN an increase in the putative DAHP synthase and EPSP synthase gene expression was observed. As previously discussed, the application of 1-MCP in combination with PHEN induces higher expression of the putative GOX and MnSOD genes (Fig. 1), therefore these treatment may be producing higher levels of ROS and thus higher DAHP synthase and EPSP synthase relative expression.

The effect of DPI, PHEN, and 1-MCP individual and combined treatments on the relative expression of secondary metabolism related genes is shown in Fig. 4. ROS have a primordial role on inducing the expression of secondary metabolism related genes (Fig. 4). Likewise, a significant decrease on the wound-induced activation of the six genes was observed on the shredded-carrots treated with water. For genes such as the phenylalanine ammonia-lyase (PAL), the putative 4-coumarate-CoA ligase (4CL) and the caffeoyl-CoA 3-*O*-methyltransferase (CCoAOMT), the application of PHEN induced higher relative expression levels, indicating that JA negatively regulate their relative expression. Likewise, the presence of ROS, ET and JA is necessary to promote the wound-induced expression of *trans*-cinnamate-4-hidroxylase (C4H, Fig. 4B). Regarding chalcone synthase 2 (CHS2) gene, the inhibition of ROS and ET induced a reduction on relative expression (Fig. 4E). In the case of the putative bergaptol *O*-methyltransferase (BOMT) gene (Fig. 4F), involved in the production of furanocumarins, ROS and ET also promote its wound-induced expression. However, this effect is only observed when JA is present in the tissue. Interestingly, all genes where ROS play a primordial role on their wound-induced activation (PAL, 4CL, and CCoAOMT) showed higher relative expression in the 1-MCP+PHEN treatment as compared with those genes where all signaling molecules are important to wound-induce their expression. This could be attributed to the higher concentration of ROS that this combination of inhibitor exerted due to the cross-talk process described earlier.

### ROS play a major role on the wound-induced accumulation of phenolic compounds in carrots

**Figure 5:**
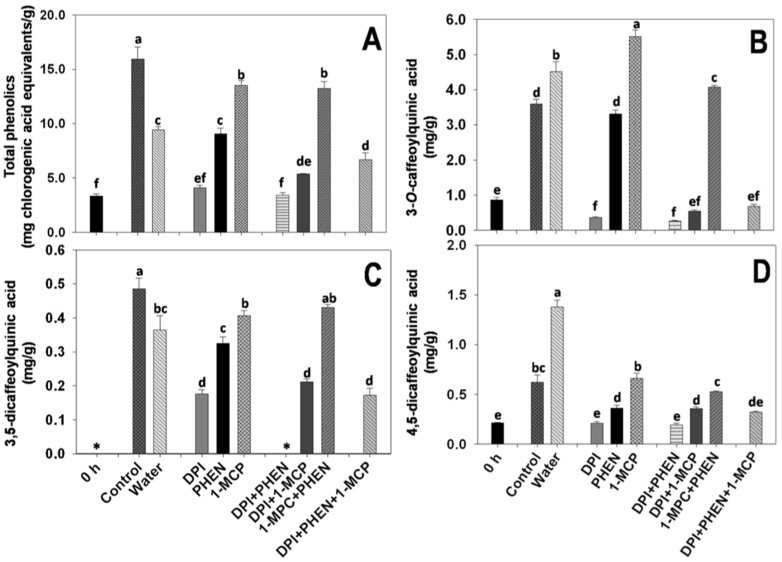
Effect of diphenyleneiodonium chloride (DPI), phenidone (PHEN) and 1-methylcyclopropene (1-MCP) on the wound-induced accumulation of total and individual phenolic compounds in carrot tissue. Total phenolic compounds (A), 3-caffeoylquinic acid (3-CQA, B); 3,5-dicaffeoylquinic acid (3,5-diCQA, C); and 4,5-dicaffeoylquinic acid (4,5-diCQA, D). Phenolic concentration was determined 48 h after wounding. Concentrations were determined based on dry weight. Data represents the mean of 4 replicates ± standard error of the mean. Bars with different letters indicate statistical difference by the LSD test (p < 0.05).

The application of wounding stress produced ~375% of increase in the total PC of carrots after 48 h of storage at 20°C as compared with wholes before storage (Fig. 5A). Washing the shredded-carrots on water induced lower production of total PC. A similar phenomenon was observed on the genes from the primary and secondary metabolism where a significant decrease on relative expression was observed as a result of submerging shreds on water (control for all submerged samples) (Figs. 2–3). These results suggest the partial removal of a primary signal that triggers the production of total PC in wounded plants, such as extracellular ATP which has been proposed as the primary signal produced at the site of injury that trigger NADPH oxidase activity and thus ROS production^5^,^21^.

Dipping the shredded-carrots on PHEN solution did not show significant reduction on total PC as compared with water controls. Therefore, JA is not directly related with the accumulation of total PC (Fig. 5). The gaseous application of 1-MCP produced a slightly reduction (p < 0.05) on the wound-induced accumulation of total PC as compared with the control. The effect of applying exogenous ET and methyl jasmonate (MJ) on PAL activity and on the accumulation of total PC in shredded-carrots has been previously studied^6^. The authors observed that the exogenous application of MJ does not affect PAL activity and total PC accumulation. Likewise, the exogenous application of ET produced higher PAL activity and total PC values^6^. In accordance with previously reported data, the wound-induced activation of PAL gene (Fig. 4A) and the accumulation of total PC (Fig. 5) was slightly lower on the 1-MCP treatment, indicating that ET plays a mild role on activating the phenylpropanoid metabolism and thus on the accumulation of total PC. The lower accumulation of total PC observed on the 1-MCP treatment may also be related with the involvement of ET on the wound-induced activation of the primary metabolism. Shreds treated with 1-MCP shower lower wound-induced activation of genes with putative function related with the biosynthesis of aromatic amino acids (DAHP synthase and EPSP synthase, Fig. 3). Therefore, the accumulation of carbon skeletons needed for phenolics biosynthesis may be lower on 1-MCP treatment.

The application of DPI in combination with either PHEN or 1-MCP inhibited the wound-induced production of total PC as compared with water controls (Fig. 5A). This data agrees with the relative expression of PAL observed for this treatment (Fig. 4A). The 1-MCP+PHEN treatment showed the highest relative expression of PAL as compared with all other treatments. As previously discussed, this higher expression of PAL may be attributed to higher ROS levels produced on this treatment because of the negative regulation that JA and ET exert on MnSOD and GOX due to the cross-talk described earlier (Fig. 2). Likewise, genes with putative function on the primary metabolism showed higher relative expression on the 1-MCP+PHEN treatment (Fig. 3), suggesting that the higher accumulation of total PC observed on this treatment, as compared with the water control, is due to a higher biosynthesis of carbon skeletons needed for PC biosynthesis.

The accumulation of individual PC was also evaluated in the DPI, PHEN and 1-MCP treatments (Figs. 5B, 5C, & 5D). Interestingly, shredded-carrots washed with water showed higher accumulation of 3-*O*-caffeoylquinic acid (3-CQA) as compared with the control (Fig. 5B). Although the wound-induced activation of genes related with the biosynthesis of hydroxycinnamic acids (PAL, C4H, and 4CL) decreased by washing the shredded-carrots on water (Figs. 4), a higher accumulation of 3-CQA in the tissue was observed (Figs. 5B). This can be explained in terms of rate of production and utilization. It is well known that wounding stress induces the production of lignin during wound healing in carrots^27^. Lignin is composed of monolignols residues that are synthesized from hydroxycinnamic acids precursors such as the caffeoylquinic acids, where 3-CQA is one of the preferred substrates^28^. Therefore, the higher accumulation of 3-CQA observed on the shreds washed with water may be related with a lower rate of utilization for lignin biosynthesis. As observed for the putative CCoAOMT gene (Fig. 4D), which is involved on lignin biosynthesis, its wound-induced activation is significantly decreased by washing the carrots with water, supporting that lignin biosynthesis is also decreased by washing shredded-carrots on water. A similar behavior was observed in the 1-MCP treated samples, where higher accumulation of 3-CQA was observed as compared with the control. Interestingly, for the 1-MCP treatment, higher 4CL relative expression levels were observed (Fig. 4C), indicating that ET down-regulates the production of *p*-coulmaroyl CoA which is a key metabolic intermediate involved on the synthesis of 3-CQA. Likewise, lower CCoAOMT gene expression levels were observed on the 1-MCP treatment, indicating that ET up-regulates lignin biosynthesis, and thus for this treatment the accumulation of 3-CQA was favored.

Furthermore, the wound-induced accumulation of 3-CQA was completely repressed on samples treated with DPI, suggesting that ROS play a key role on the wound-induced production of this PC, which is in agreement with the lower expression levels observed for all secondary metabolites related genes in DPI treated samples. On the other hand, PHEN produced slightly lower concentrations of 3-CQA in the tissue as compared with the water control. Since 3-CQA is one of the preferred substrates for lignin biosynthesis, and JA down regulates the expression of CCoAOMT (Fig. 4D), it is likely that the rate of lignin biosynthesis is increased in PHEN treated samples, and thus lower 3-CQA accumulation was observed as compared with water controls, where the expression of CCoAOMT gene is down-regulated. Likewise, the 1-MCP+PHEN treatment showed a lower concentration of 3-CQA as compared with the water control, which may be related with a higher rate of lignin biosynthesis (CCoAOMT gene, Fig. 4D) due to higher levels of ROS that this combination of inhibitor exerted due the cross-talk described earlier (Fig. 2).

Regarding the 3,5-diCQA and 4,5-diCQA, the application of DPI alone or in combination with 1-MCP and/or PHEN on shredded-carrots inhibited their wound-induced accumulation (Figs. 5C and 5D). The inhibition exerted by DPI was more evident on 4,5-diCQA (Fig. 5D), where no accumulation was observed, whereas for the 3,5-diCQA slight increases were observed for all treatments because the compound was not detected on time zero samples (Fig. 5C). The biosynthetic pathway of 3,5-diCQA and 4,5-diCQA is not completely elucidated, however it is known that both compounds are produced from 3-CQA. Therefore, it is likely that the conversion of 3-CQA to its derivatives (3,5-diCQA and 4,5-diCQA) may depend on 3-CQA availability. However, further experimental work evaluating the effect of inhibitors on the expression of genes related with the conversion of 3-CQA into 3,5-diCQA and 4,5-diCQA would be needed to better understand how the production of these derivatives are mediated by ET, ROS and/or JA.

**Figure 6:**
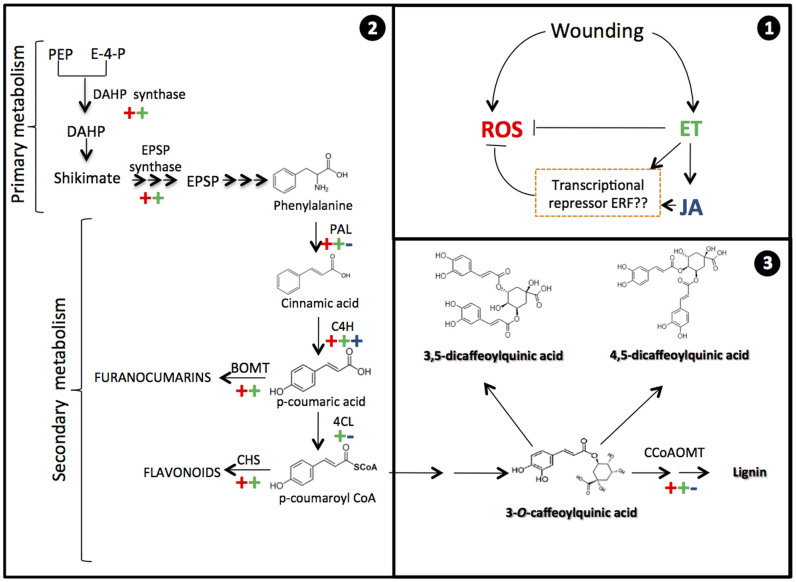
Down (−) and up-regulation (+) of primary and secondary metabolism related genes as well as secondary metabolites production by reactive oxygen species (ROS), ethylene (ET), and jasmonic acid (JA). ROS and ET are generated few minutes after wounding stress (1). ET production induces the production of JA. The primary role of ET and JA is to modulate ROS levels. ROS act as signaling molecules to activate the primary and secondary metabolism of carrots (2). The production of secondary metabolites (3) which occur after the production of signals (1) and the activation of the primary and secondary metabolism (2) is mainly mediated by ROS. However, ET and JA play a mild role on modulating the expression of certain genes as well as on the accumulation of secondary metabolites (3).

The data obtained as a result of the present research work allowed the generation of relevant and novel scientific information regarding the role of ROS, ET and JA on the wound-response of carrot tissue. The information generated in this research is summarized in Fig. 6. Data suggests that upon the application of wounding stress ROS and ET are simultaneously produced, and ET induces JA biosynthesis. ET and JA play a key role on modulating ROS levels. After the signaling molecules are produced, the primary and secondary metabolisms are activated on the wounded-carrots. Likewise, secondary metabolites are produced as a result of primary and secondary metabolism elicitation. ROS play the major role on the activation of the primary and secondary metabolism as well as on the accumulation of secondary metabolites, whereas JA and ET play an important role on controlling ROS levels and on the activation of certain genes. Additionally, JA and ET affect the accumulation of phenolic compounds by modulating their rate of synthesis and their rate of utilization for lignin biosynthesis. The information generated in this investigation opens the possibility of envisage strategies to manipulate the production of phenolic compounds with a wide range of applications on the functional foods, dietary supplements, cosmetics and agrochemical industries.

## Methods

### Reagents

Folin-Ciocalteu reagent, sodium carbonate (Na_2_CO_3_), diphenyleneiodonium chloride (DPI), phenidone (PHEN), methanol (HPLC grade), and acetonitrile (HPLC grade), were purchased from Sigma Chemical Co. (St. Louis, MO, USA). Chlorogenic acid was purchased from Fisher Scientific (Houston, TX, USA). 1-methylcyclopropene (1-MCP) powder (SmartFresh^TM^) was kindly provided by AgroFresh^Inc.^ (Springhouse, PA, USA).

### Generation of subtractive cDNA library

#### RNA extraction, isolation of poly A^+^ mRNA and cDNA synthesis

Total RNA from time 0 h samples and time 24 h-shreds was isolated following the hot borate method^29^. Isolation of poly A^+^ mRNA from total RNA was performed using the Oligotex mRNA Spin-Column kit (Qiagen). Purified poly A^+^ mRNA from time 0 h samples and time 24 h-shreds were used for cDNA synthesis by the PCR-Select^TM^ cDNA subtraction kit (Clontech).

#### Suppression subtractive hybridization (SSH) and generation of cDNA library

SSH was conducted using PCR-Select^TM^ cDNA subtraction kit (Clontech). Forward and reverse cDNA libraries were generated. Forward SSH was performed using cDNA from time 24-h shreds samples as tester and cDNA from time 0 h-wholes as driver. For the reverse SSH, 0 h-wholes and 24 h-shreds samples were used as tester and driver, respectively. Briefly, the tester and driver cDNAs were digested with the restriction enzyme RsaI (provided in the kit) to obtain short, blunt-ended fragments. The tester was divided in two populations. One population was ligated to adaptor 1 and the other to adaptor 2R (both provided in the kit). Each tester was then hybridized with excess driver using a driver/tester ratio of 2:1 (v/v). The two reactions were mixed together for a second SSH. Fragments in the tester (time 24 h-shreds) cDNA, but not in the driver (time 0 h-wholes) were specifically amplified in two PCRs (25 cycles of primary PCR and 12 cycles of secondary PCR) with the advantage cDNA polymerase mix (provided in the kit). qRT-PCR analysis was performed to estimate the efficiency of subtraction by comparing the cycle threshold (Ct) of α-tubulin before and after subtraction. qRT-PCR reactions were carried out using α-tubulin specific primers (forward primer 5′-CTTGCTGGGAACTTTACTGCCT-3′, reverse primer 5′- CAAAGATTGCACGAGGTACATGC-3′). The Ct values for α-tubulin transcript using the subtracted and unsubtracted cDNAs as templates were 38 and 23, respectively, indicating that the subtraction was carried out appropriately.

An aliquot of 10 μl of secondary PCR reaction from the subtracted cDNA sequences was purified using a S.N.A.P.^TM^ column kit (Invitrogen), and 3 μl of the purified secondary PCR reaction was inserted into the T/A cloning vector pGEM-T Easy (Promega) and cloned into One Shot® TOP10 chemically competent *Escherichia coli* (Invitrogen) following manufacturer's recommendations. Individual transformants carrying cDNA fragments were isolated from white colonies on X-gal agar plates and placed in an arrayed 96-well format. Plasmid DNA preparations and sequencing were performed for the obtained clones from the forward library (Macrogen Corp, Rockville, MD, USA).

#### Sequence analysis and functional classification

All the generated sequences were compared to the NCBI database using the Basic Local Alignment Search Tool (BLAST; http://www.ncbi.nlm.nih.gov/BLAST/). The clones of genes were classified according to their metabolic function into seven functional categories, as genes related to stress-signaling pathways, primary metabolism genes, secondary metabolites biosynthesis genes, other disease and stress response genes, other metabolic genes, novel genes, and unidentified function genes.

#### Analysis of differential expression of genes by real-time quantitative reverse transcription PCR (qRT-PCR)

To confirm the differential expression of genes involved on stress-signaling pathways as well as on the primary and secondary metabolism, a qRT-PCR approach was used. Total RNA from time 0 h samples and time 24 h-shreds were separately isolated following the hot borate method^29^. RNA quality was determined spectrophotometrically measuring the absorbance values at 260 and 280 and calculating the 260/280 ratio. RNA quantity was determined spectrophotometrically at 260 nm. The RNA integrity was evaluated on 1% (w/v) agarose gels. Total RNA was treated with DNAse using the RNase-Free DNase Set (Qiagen) and cleaned up using the RNeasy Mini Kit (Qiagen) following manufacture's recommendations. First strand of cDNA was obtained by reverse transcription reaction with 2 μg of total RNA as template, using SuperScript™ III Reverse Transcriptase (Invitrogen) and random hexamers (IDT), according to standard procedures. The single stranded cDNA obtained was subjected to qRT-PCR. Quantitative RT-PCR was performed with the ABI prism 7900 HT Sequence Detection System (Applied Biosystems) using Power SYBR® Green PCR Master Mix (Applied Biosystems) and gene-specific primers (IDT) that were designed using the Primer Premier 5.0 software (Premier Biosoft International) (Table S1–S4). Conditions, procedures and analyses for qRT-PCR were performed as described by Salzman *et al.*^30^.

qRT-PCR experiments were performed by triplicate and the expression values were normalized against α-tubulin. To test whether α-tubulin behaved as a housekeeping gene, cDNA samples from wounded and non-wounded carrots analyzed by qPCR were synthesized with dap spike mRNA (obtained from the ATCC; number 87486) added as internal control (0.01%)^31^. For each cDNA, transcript abundances of α-tubulin and dap were analyzed by qPCR and the ratios of dap to α-tubulin were calculated for all samples. Results demonstrated that abundance of α-tubulin remains unaltered between samples. Amplification specificity was determined by dissociation curve analysis, and the amplification product sizes were confirmed in an agarose gel to ensure the absence of non-specific PCR products.

The relative expression of the genes were calculated as 2^^∧^^(ΔΔCt), where:
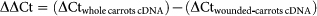

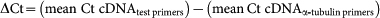


### Pharmacological studies

Shredded-carrots used for the pharmacological studies were prepared and stored as previously described. Two independent experiments were performed. On the first experiment, the effect of wounding stress on the relative expression of all the genes under investigation was evaluated through time in order to determine if the genes had an early or late response after wounding stress. On the second experiment, inhibitors of ET, JA, and ROS were applied individually and in combination on the wounded-carrot tissue and the time at which the relative expression of each gene was evaluated was selected based on the first experiment.

To block ET action, 12 h prior to wounding whole carrots stored at 10°C were exposed to headspace vapors (2,000 ppb = 2.0 μl/L) of 1-MCP produced with 1-MCP powder (SmartFresh^TM^) containing 0.14% (w/w) of active ingredient. After the application of wounding, the shredded-carrots were stored at 20°C for 48 h in 4 L glass jars. Samples were collected at 0, 0.5, 6, 12, 24, and 48 h after wounding. Each time the jars were opened either for sampling or for ventilation (to avoid CO_2_ accumulation >0.5%) 1-MCP was applied on the jars to adjust 1-MCP headspace vapors concentration (2,000 ppb). Samples exposed to air were used as controls for the 1-MCP treatments.

Treatments using DPI and PHEN were performed to inhibit ROS and JA biosynthesis, respectively. Both inhibitors (DPI and PHEN) were applied by dipping the shredded-carrots for 2 min in solutions containing the inhibitors. DPI solutions (315 μM) were prepared in 1% DMSO, whereas PHEN solutions (2000 μM) were prepared in 1% ethanol^20^. Samples were allowed to dry during few minutes and placed inside glass jars where the treatments were stored for at 20°C for 48 h. As indicated for the 1-MCP treatments, samples were collected at 0, 0.5, 6, 12, 24, and 48 h after wounding. Likewise combinatory treatments (DPI+PHEN, DPI+1-MCP, 1-MCP+PHEN, and DPI+PHEN+1-MCP) were prepared, stored, and sampled as previously mentioned. For all treatments involving DPI and/or PHEN shredded-carrot tissue dipped for 2 min on nanopure water was used as control.

RNA extraction, cDNA synthesis and qRT-PCR analyses were performed as described in previous sections of the manuscript. The sequences for primers are shown in table S6. With the exception of MnSOD and NADPH oxidase, all primers for qRT-PCR analysis were designed based on sequences from the wound-induced subtractive cDNA library (tables S1–S4). For NADPH oxidase gene, a sequence from a deep-coverage carrot bacterial artificial chromosomal (BAC) library (GenBank: FJ147918.1), showed similarity to the NADPH oxidase gene from *Solanum tuberosum* (*StrbohC*, GenBank: AB198716.2) and *Nicotiana langsdorffii x Nicotiana sanderae* (*rbohD*, GenBank: DQ497543.1). The portion of the carrot sequence (GenBank: FJ147918.1) that showed similarity to the NADPH oxidase gene from *Solanum tuberosum* and *Nicotiana langsdorffii x Nicotiana sanderae* was used to design specific primers (forward primer 5′-AGTGGGCAATACATGTTTGTCAAA-3′, reverse primer 5′-CATGGGTGTTGCCCCTATGCC -3′) to further amplify by PCR a putative NADPH sequence from carrots. PCR products were analyzed by agarose gel electrophoresis, and a band was selected based to the expected fragment size. The selected band was purified using the QIAquick gel extraction kit (Qiagen) and sent for sequencing analysis at the Institute for Plant Genomics and Biotechnology from Texas A&M University (College Station, TX, USA). The obtained sequence was used to design specific primers for qRT-PCR analysis as shown in table S6. To obtain a putative MnSOD sequence from carrots, degenerate primers were designed (forward primer 5′-GATTATGCAGATACACCACCAGAARCAYCAYCA-3′, reverse primer 5′-ACACTCTTTTTCATATACTTCAGAAGCATAYTTCCARTT-3′) based on conserved protein regions of MnSOD from *Pisum sativum*, *Glycine max*, *Prunus persica* and *Capsicum annum*. As described for NADPH oxidase, PCR products were analyzed by agarose gel electrophoresis, the selected band was purified using the QIAquick gel extraction kit (Qiagen) and sent for sequencing analysis. The obtained sequence was used to design specific primers for qRT-PCR analysis as shown in table S6.

### Sample preparation for phytochemical analyses

Prior to phytochemical analyses, shredded-carrots were lyophilized using a freeze-drying system (FTS Systems,Inc., Stone Ridge, NY) at −50°C and 200 mmHg of pressure. Freeze-dried carrot powder (~250 mg) was homogenized with methanol (20 mL) using an Ultra-Turrax homogenizer (IKA Works, Inc., Wilmington, NC) and centrifuged at 29,000 × *g* for 15 min at 4°C. The clear supernatant (methanolic extract) was used for the analyses of total soluble phenolics (PC) and for the identification and quantification of the individual phenolic compounds by HPLC-PDA and HPLC-ESI-MS^n^. The methanolic extracts were passed through nylon membranes (0.2 μm) prior to injection to the chromatographic systems.

### Analysis of total soluble phenolics (PC)

Total soluble phenolics were determined using the method described by Swain and Hillis^32^. Methanolic extracts (15 μL) were diluted with nanopure water (240 μL) in a 96-well microplate well, followed by the addition of 0.25N Folin-Ciocalteu reagent (15 μL). The mixture was incubated for 3 min and then, 1N Na_2_CO_3_ (30 μL) was added. The final mixture was incubated for 2 h at room temperature in the dark. Spectrophotometric readings at 725 nm were collected using a plate reader (Synergy HT, Bio-Tek Instruments, Inc., Winooski, VT). Total phenolics were expressed as mg chlorogenic acid equivalents/g of freeze-dried carrot powder.

### Identification and quantification of phenolic compounds by HPLC-DAD and HPLC-ESI-MS^n^

The HPLC system was composed of two 515 binary pumps, a 717-plus autosampler, and a 996-photodiode array detector (Waters Corp., Mildford, MA). Phenolic compounds were separated on a 4.6 mm × 150 mm, C-18 reverse-phase Atlantis column (Waters Corp., Mildford, MA) that was maintained at 40°C using a SpectraPhysics SP8792 column heater. The mobile phases consisted of water/formic acid 1% (99:1, v/v, phase A) and acetonitrile (phase B). The gradient solvent system and data processing was performed as described by Jacobo-Velázquez et al.^5^ Mass spectrometric analyses were performed on a Thermo Finnigan LCQ Deca XP Max Ms*^n^* ion trap mass spectrometer equipped with an ESI ion source (ThermoFinnigan, San Jose, CA). Separations were conducted using the Phenomenex (Torrace, CA) Synergi 4 μ Hydro-RP 80A (2 × 150 mm) with a C18 ward column. Mobile phases consisted of water/formic acid 1% (99:1, v/v, phase A) and acetonitrile (phase B) at a flow rate of 200 μL/min. The gradient solvent system and electrospray ionization conditions were as described by Jacobo-Velázquez et al.^5^. The identification of individual phenolics was based on their PDA spectra and ESI-MS^−^ fragmentation patterns as compared with authentic standards or based on previously reported data^33^.

### Statistical analysis

Analyses were performed using four repetitions. Data represent the mean values of samples and bars indicate their standard error. Analyses of variance were conducted using JMP software version 5.0 (SAS Institute Inc. Cary, NC, USA) and mean separation performed using the LSD test (p < 0.05).

## Supplementary Material

Supplementary Information Supplementary Figures and Tables
