# Supplementary material for: Cross-talk between signaling pathways: The link between plant secondary metabolite production and wounding stress response
Source: Sci Rep. 2015 Feb 25;5:8608. doi: 10.1038/srep08608 (PMC5390084; doi:10.1038/srep08608)
Supplement: Supplementary Information — Supplementary Figures and Tables [file srep08608-s1.pdf]

Supplementary Online Material

Cross-talk between signaling pathways: The link between plant secondary metabolite production and wounding stress response

Daniel A. Jacobo-Velázquez, Mauricio González-Agüero & Luis Cisneros-Zevallos

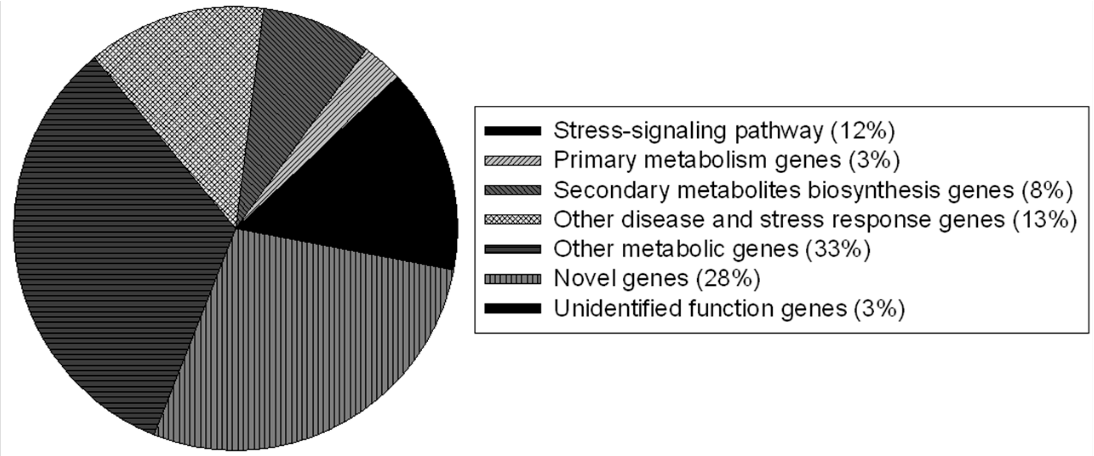

Fig. S1. Functional classification of genes differentially expressed in carrots after wounding stress.

Table S1. Genes with putative function involved on the biosynthesis and signaling of ethylene, jasmonic acid and reactive oxygen species activated by wounding stress in carrots.

Sequence ID [(i)]	Size (bp)	Gene identification	Accession n°	E-value	EST redundancy for a gene (%)	Sequence for forward (F) and reverse (R) primers (5'→3')	Relative expression [(ii)]
Ethylene signaling genes							
2401-G1	418	Ethylene-responsive transcription factor 1B, putative [*Ricinus communis*, XM_002531493]	In process	$1x10^{-99}$	0.10	F: TGGCTCTGAAGAAGAAGCATTCG<br>R: CAGCACCTAAGTCCTCAAACACA	107.84±22.34
Ethylene biosynthesis genes							
2408-C2	1175	S-adenosyl-L-methionine synthetase 1 [*Daucus carota*, AY583461]	Already registered	0.0	0.20	F: GCAACAACAGGTTCCTGAAGAC<br>R: TCCCACTTGAGGGGCTTGACTA	21.04±4.32
2402-D6	699	1-aminocyclopropane-1-carboxylate synthase [*Carica papaya*, AM113989]	In process	$1x10^{-59}$	0.93	F: GCTGTTTCTCGCTAATTTGCTG<br>R: ACTGGAAGAAATCGGCATCAAG	3.86±0.78
Jasmonic acid biosynthesis genes							
2405-E7	520	Lipoxygenase 2 [*Actinidia deliciosa*, DQ497797]	In process	$4x10^{-128}$	0.20	F: TTAAGAGGGGAATGGCAGTTGAG<br>R: GTCTTGTAGTAGAAGCCGCAGTA	556.43±61.18
2401-F12	374	Lipoxygenase 5 [*Actinidia deliciosa*, DQ497796]	In process	$1x10^{-58}$	0.20	F: CGACCCTGATGGCGTTTACCT<br>R: TCCAATCTGGGATGTCTCGTTGT	498.17±58.91
2401-H7	495	12-oxophytodienoate reductase, putative [*Vitis vinifera*, XM_002281083]	In process	$4x10^{-116}$	1.34	F: GGATGAAAAGTGTAGGAGAGAAG<br>R: CGTAACCCCCAGCAACAATAAA	197.04±26.24
2406-E9	383	Omega-6 fatty acid desaturase [*Petroselinum crispum*, U86072]	In process	$2x10^{-157}$	1.23	F: ACAACTCATCGGAATGGGATTGG<br>R: AGTATCAGTGATGTTATGGAGCACC	46.34±9.75

Table S1. continued.

	Size (bp)	Gene identification	Accession n°	E-value	EST redundancy for a gene (%)	Sequence for forward (F) and reverse (R) primers (5'→3')	Relative expression [ii]
Jasmonic acid biosynthesis genes							
2406-F4	897	3-ketoacyl-CoA thiolase B [*Mangifera indica*, X75329]	In process	0.0	1.13	F: CATTTAAGAAGGATGGGACCACA<br>R: GCGGCAAAACTCCTGAATACACC	35.96±2.47
2406-H7	768	Fatty acid desaturase/hydroxylase [*Daucus carota*, AF349965]	Already registered	0.0	0.62	F: CTTGAGAACGACGAGGTTTATGT<br>R: CATTGAACATCAGATACAGAGGGA	18.48±5.74
Reactive oxygen species biosynthesis genes							
2401-D7	555	NADH-ubiquinone oxidoreductase, putative [*Prunus armeniaca*, AF139496]	In process	$6x10^{-146}$	2.05	F: GTGCCAGAAACACTGCCAGAG<br>R: AAGATCAATCCATCCGCCTCAG	523.27±43.24
2406-C9	265	Glycolate oxidase, putative [*Vitis vinifera*, XM_002278068]	In process	$1x10^{-61}$	0.30	F: TGATGGTGAGGCTGGTGTGAGT<br>R: GTGTGATCTCTTTGATTGAGCGG	27.9±0.48
Reactive oxygen species modulation/detoxification genes							
2401-B1	912	NADPH:quinone reductase, putative [*Vitis vinifera*, XM_002262940]	In process	$2x10^{-85}$	1.44	F: TTTGAGGAGTTGCCTGAGAAGT<br>R: ATGTGACACCAGGTTCAGTAAC	123.81±33.45
2410-E5	509	Glutathione-s-transferase omega, putative [*Ricinus communis*, XM_002525158]	In process	$1x10^{-72}$	2.88	F: CCAGATGACCCTGCTAAAAGAGA<br>R: GAGAGCCGTTTCCATGTAATCAA	113.98±11.43
2402-A6	559	Peroxidase [*Panax ginseng*, AB232686]	In process	$2x10^{-102}$	0.62	F: GCAAGTGGACCTAGCGAGAAAA<br>R: CCGCAAGAGCAGTAATATCAGC	34.10±4.81
2405-E8	653	Anionic peroxidase [*Petroselinum crispum*, L36981]	In process	0.0	0.41	F: TGGTAGGGTTGTTTCTTGTGCTG<br>R: GTTGGCTAAGGTTGCTTCTGTTG	30.94±4.29

Table S1. continued.

Sequence ID [i]	Size (bp)	Gene identification	Accession n°	E-value	EST redundancy for a gene (%)	Sequence for forward (F) and reverse (R) primers (5'→3')	Relative expression [ii]
Reactive oxygen species modulation/detoxification genes							
2402-D7	561	Glutathione S-transferase [*Petroselinum crispum*, AF177944]	In process	$6x10^{-140}$	1.13	F: GTATCACATTCCTGGACAAGACC<br>R: TTCTCAGAAGTTGGCAATACACC	28.72±9.63
2407-D2	161	Glutathione peroxidase [*Litchi chinensis*, FJ172343]	In process	$9x10^{-42}$	0.10	F: AAAGGTGGACTCTTTGGTGATGG<br>R: AATGCTGAGAGGAGAGGTTGTG	26.95±4.52
2406-D3	286	Thioredoxin H-type 1, putative [*Nicotiana benthamiana*, GQ354821]	In process	$8x10^{-59}$	0.20	F: TGCTAAGAAGATGCCTCATGTCA<br>R: CAACCCTGTCCACAATTATCCCT	5.71±0.94
2404-E6	531	Peroxiredoxin, putative [*Vitis vinifera*, XM_002283616]	In process	$3x10^{-105}$	0.10	F: TTTGTGATGAAGGCGTGGAAGG<br>R: CAAATCAAGCTCAACCCCGATAG	5.45±0.92

(i) Identification number on the SSH library.

(ii) Data represents the mean of three replicates ± standard error of the mean.

Table S2. Genes with putative function related with the primary metabolism up-regulated by wounding stress in carrots.

Sequence ID [(i)]	Size (bp)	Gene identification	Accession n°	E-value	EST redundancy for a gene (%)	Sequence for forward (F) and reverse (R) primers (5'→3')	Relative expression [(ii)]
Glycolisis and pentose phosphate pathway genes							
2410-E8	518	6-phosphogluconate dehydrogenase, putative [*Populus trichocarpa,* XM_002311387]	In process	$8x10^{-170}$	0.51	F: GCCCTTGTGTTACATACATTGGT<br>R: AGTTCCTCGTTTGAGAGTTTACC	14.29±2.45
2408-A11	269	Fructose-bisphosphate aldolase, putative [*Ricinucs communis,* XM_002528547]	In process	$6x10^{-66}$	0.20	F: AGTTCCCACTGTTGTGTTTTTGTC<br>R: ACTCCATGACTTCTTTGCCTTCA	6.52±1.36
2409-F3	354	Cofactor-independent phosphoglyceromutase [*Apium graveolens*, AJ132256]	In process	$1x10^{-153}$	0.10	F: GGCGTATGTATGTTACAATGGAC<br>R: TAGCCTTGGGTTCTGCCCTTAG	5.49±0.33
Shikimate pathway genes							
2401-F9	927	3-deoxy-D-arabino-heptulosonate [*Petroselinum crispum*, AF012864]	In process	0.0	0.82	F: GATGCTATCAGGGCTGAAGTCAA<br>R: CCTAGATCATCAAAAGTCACGGTTC	35.29±4.61
2410-E2	733	5-enolpyruvylshikimate 3-phosphate synthase [*Camptotheca acuminata*, AY639815]	In process	0.0	0.31	F: TAGGAGCAACAGTTGAAGAGGGT<br>R: ATCTGAACAGGCAGCAAGAGAGA	25.78±1.04
Aromatic amino acid biosynthesis genes							
243-C5	459	Chorismate mutase-prephenate dehydratase, putative [*Vitis vinifera,* XM_002268665]	In process	$2x10^{-82}$	0.20	F: CGAGCACACGTTGACGAAGCTA<br>R: AAATTCCGCAGCTCCAGCAGT	29.87±4.42

(i) Identification number on the SSH library.

(ii) Data represents the mean of three replicates ± standard error of the mean.

Table S3. Genes with putative function related with the biosynthesis of phenolic compounds up-regulated by wounding stress in carrot.

Sequence ID [(i)]	Size (bp)	Gene identification	Accession n°	E-value	EST redundancy for a gene (%)	Sequence for forward (F) and reverse (R) primers (5'→3')	Relative expression[(ii)]
Hydroxycinnamic acids biosynthesis genes							
2407-H10	733	4-coumarate-coa ligase [*Populus trichocarpa*, XM_002304328]	In process	$6x10^{-97}$	1.95	F: GGGACTACTGGGACTTCCAAAG<br>R: AGCCAAATAAACCATCTCCCAGC	2174.63±246.2
2411-C9	866	Phenylalanine ammonia-lyase (gDcPAL1) [*Daucus carota*, D85850]	Already registered	0.0	1.74	F: AGGGCAAACCCGAATTTACTGAC<br>R: CATAAGAGCTTCCATCCAAGATG	65.37±2.09
2405-D9	392	Trans-cinnamate 4-monooxygenase [*Petroselinum crispum*, L38898]	In process	$1x10^{-153}$	1.85	F: CCTGGAATCATCCTTGCATTGC<br>R: TGTCCTCCTTTCTCTGCTGTGT	41.26±4.75
Flavonoids biosynthesis genes							
2406-D5	364	Hydroxycinnamoyl-CoA:anthocyanin 3-O-glucoside-6"-O-hydroxycinnamoyltransferase [*Petunia x hybrida*, AB026495]	In process	$7x10^{-48}$	0.10	F: GTTTGATGGGATGGTGTTTTTGC<br>R: CCTTGTCTGCCTCTAGTTTCTTC	113.32±23.89
2408-E6	432	Favone synthase I [*Daucus carota*, AY817675]	Already registered	0.0	0.10	F: TCACGGTATTGACAGCGGTTTG<br>R: TCACCCTGAAGATGAGTGGAGA	15.25±2.98
Lignin biosynthesis genes							
2407-F2	602	Cinnamoyl-CoA reductase [*Codonopsis lanceolata*, AB243011]	In process	$6x10^{-155}$	0.82	F: AATCCCTCAGCAGCAGGAAGATA<br>R: TTGGTAGGCACGGGGTATTCAG	609.15±152.24
2409-D9	513	Caffeoyl-CoA 3-O-methyltransferase [*Petroselinum crispum*, M69184]	In process	0.0	0.31	F: TATTCTCTCCTTGCCACTGCTCT<br>R: CCCTCTCTGAAGTCAATTTTGTG	27.10±4.68
2405-F5	755	Cinnamyl alcohol dehydrogenase [*Aralia cordata*, D13991]	In process	0.0	0.82	F: GGAGGCAATGGATCATCTTGGT<br>R: GAGAGGTAAGGTTCGAGTGGGT	16.87±4.72

(i) Identification number on the SSH library.

(ii) Data represents the mean of three replicates ± standard error of the mean.

Table S4. Genes with putative function related with the biosynthesis of secondary metabolites with health promoting properties up-regulated by wounding stress in carrots.

Sequence ID [i]	Size (bp)	Gene identification	Accession n°	E-value	EST redundancy for a gene (%)	Sequence for forward (F) and reverse (R) primers (5'→3')	Relative expression [ii]
Furanocoumarin biosynthesis genes							
2410-D12	393	Bergaptol O-methyltransferase [*Glehnia littoralis*, AB363638]	In process	$5x10^{-144}$	3.08	F: CTATGGTTCAACGCATCCTCC<br>R: TGTCCAGCAATAGAGCCACTC	12.71±5.26
Alkaloids biosynthesis gene							
2407-H8	711	Berberine bridge, putative [*Populus trichocarpa*, XM_002333922]	In process	$6x10^{-116}$	1.13	F: TGGGAGAAGACTTATTTTGGGCTA<br>R: AAACAGTGACGGTAGATGGGACA	
2410-F2	369	Reticuline oxidase precursor, putative [*Ricinus communis*, XM_002523118]	In process	$3x10^{-71}$	0.51	F: CAGGCTGCTGTAGTATGCGTGA<br>R: CAGATCAACCACAACAAACGGTG	

(i) Identification number on the SSH library.

(ii) Data represents the mean of three replicates ± standard error of the mean.

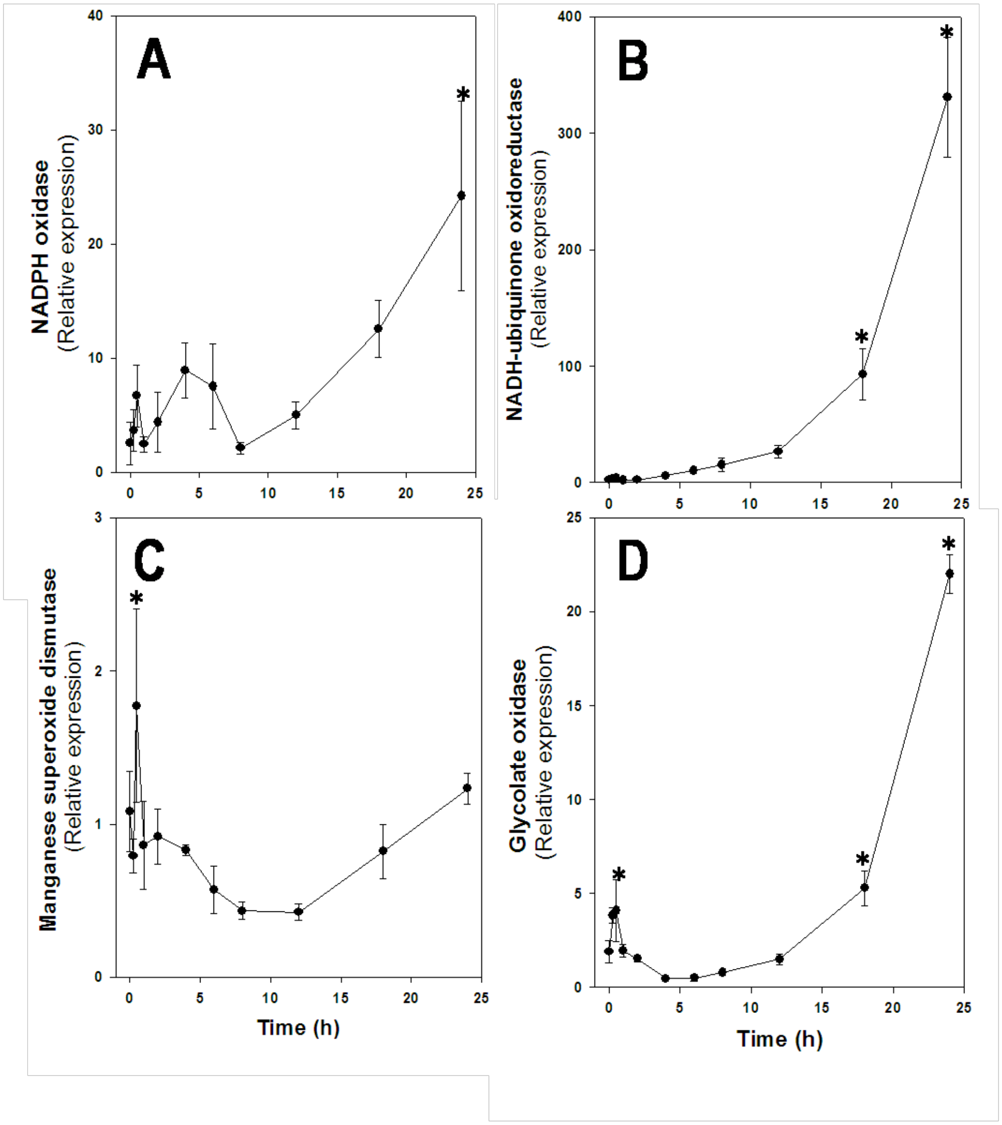

Fig. S2. Relative expression through time of genes with putative function related with the biosynthesis of reactive oxygen species (ROS) in wounded carrots. NADPH oxidase (A); NADH-ubiquinone oxidoreductase (B); Manganese superoxide dismutase (MnSOD, C); Glycolate oxidase (GOX, D). Data represents the mean of 4 replicates ± standard error of the mean. Data points with an asterisk (*) indicate statistical difference by the t-test ($p < 0.05$) with respect the relative expression of time 0 h samples.

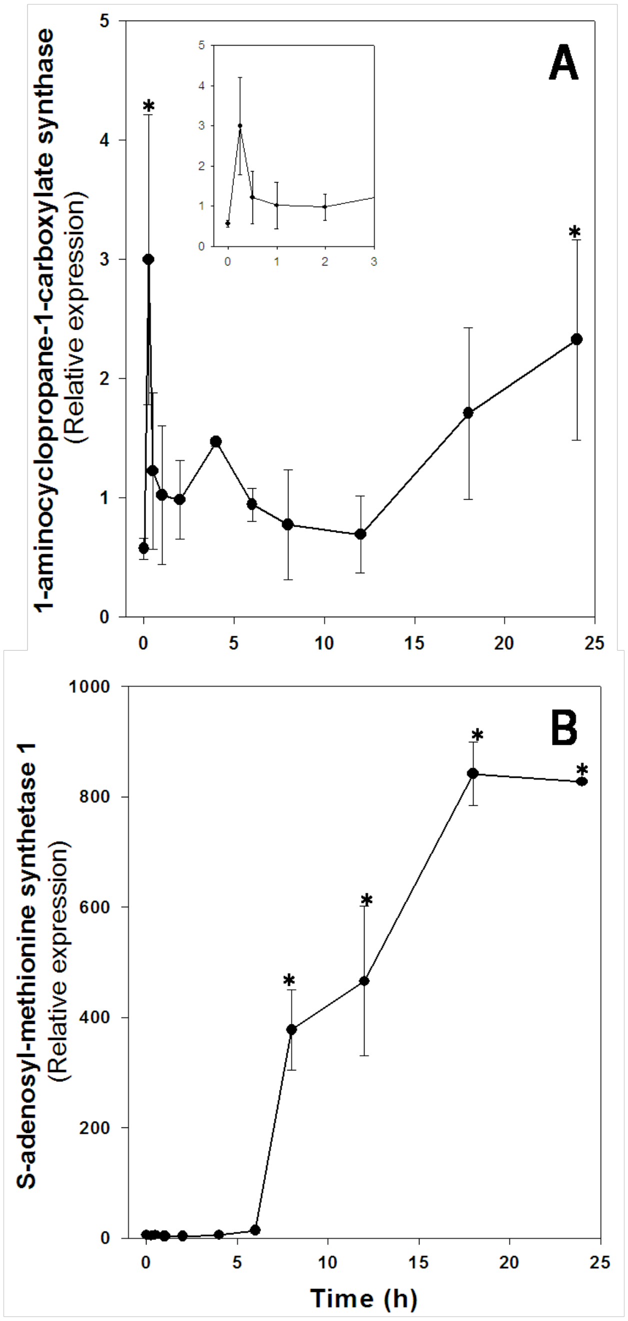

Fig. S3. Relative expression through time of genes related with the biosynthesis of ethylene (ET) in wounded carrots. 1-aminocyclopropane-1-carboxylate synthase (ACC synthase, A); S-adenosyl-methionine synthetase 1 (SAM synthetase, B). Data represents the mean of 4 replicates ± standard error of the mean. Data points with an asterisk (*) indicate statistical difference by the t-test ($p < 0.05$) with respect the relative expression of time 0 h samples.

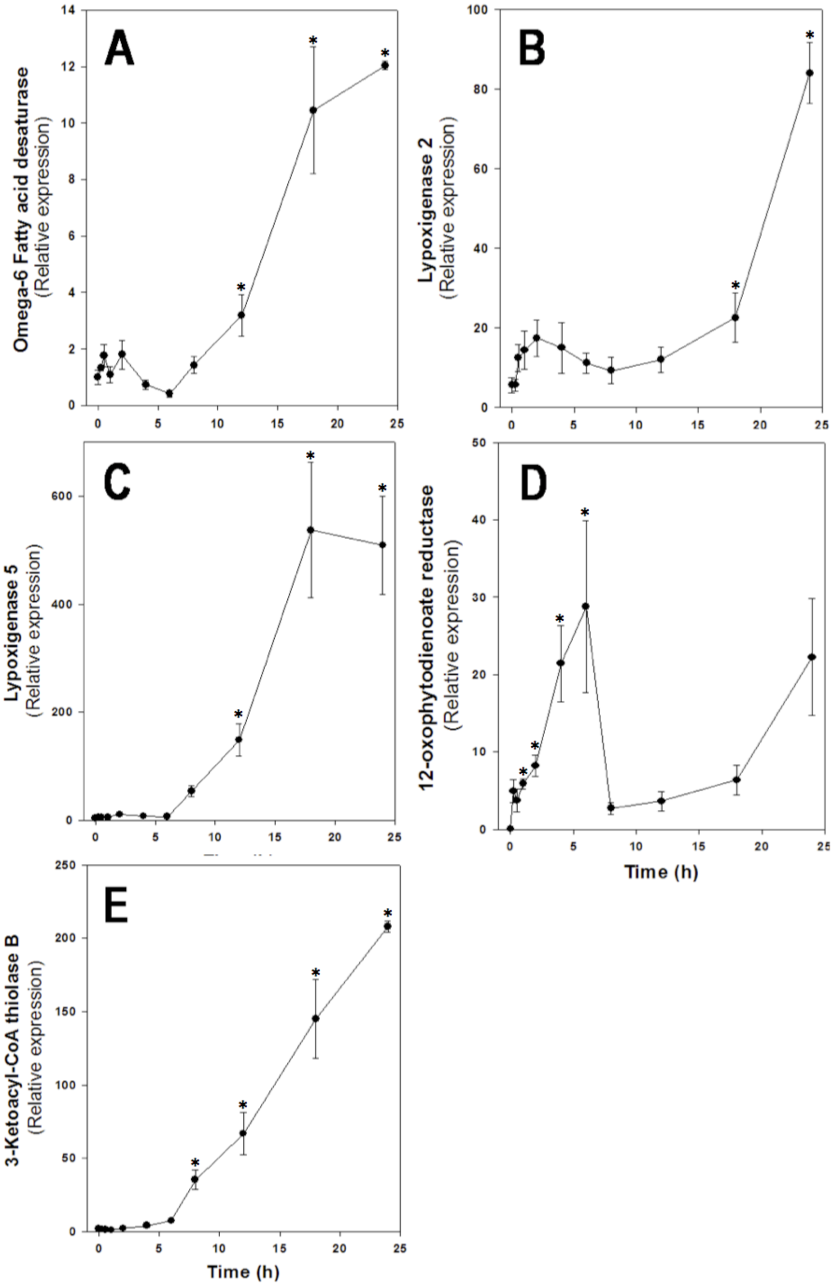

Fig. S4. Relative expression through time of genes with putative function related with the biosynthesis of jasmonic acid (JA) in wounded carrots. Omega 6-fatty acid desaturase (A); lipoxygenase 2 (LOX 2, B); lipoxyngeanse 5 (LOX 5, C); 12-oxo phytodienoate reductase (12-OPDA reductase D); 3-Ketoacyl-CoA thiolase B (3-KCT, E). Data represents the mean of 4 replicates ± standard error of the mean. Data points with an asterisk (*) indicate statistical difference by the t-test ($p < 0.05$) with respect the relative expression of time 0 h samples.

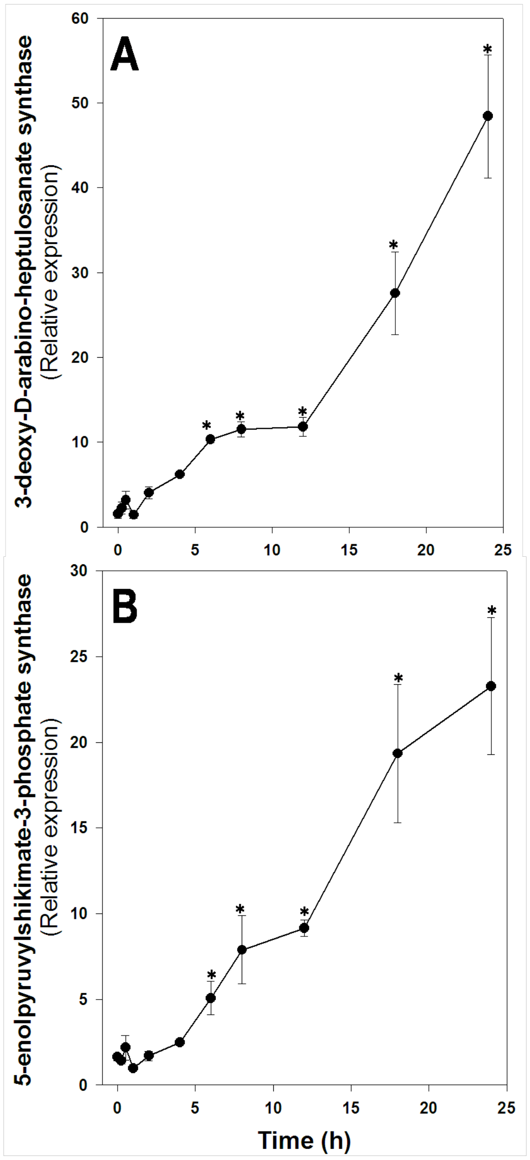

Fig. S5. Relative expression through time of genes with putative function related with the primary metabolism in wounded carrots. 3-deoxy-D-arabino-heptulosanate synthase (DAHP synthase, A); 5-enolpyrovylshikimate 3-phosphate synthase (EPSP synthase, B). Data represents the mean of 4 replicates ± standard error of the mean. Data points with an asterisk (*) indicate statistical difference by the t-test ($p < 0.05$) with respect the relative expression of time 0 h samples.

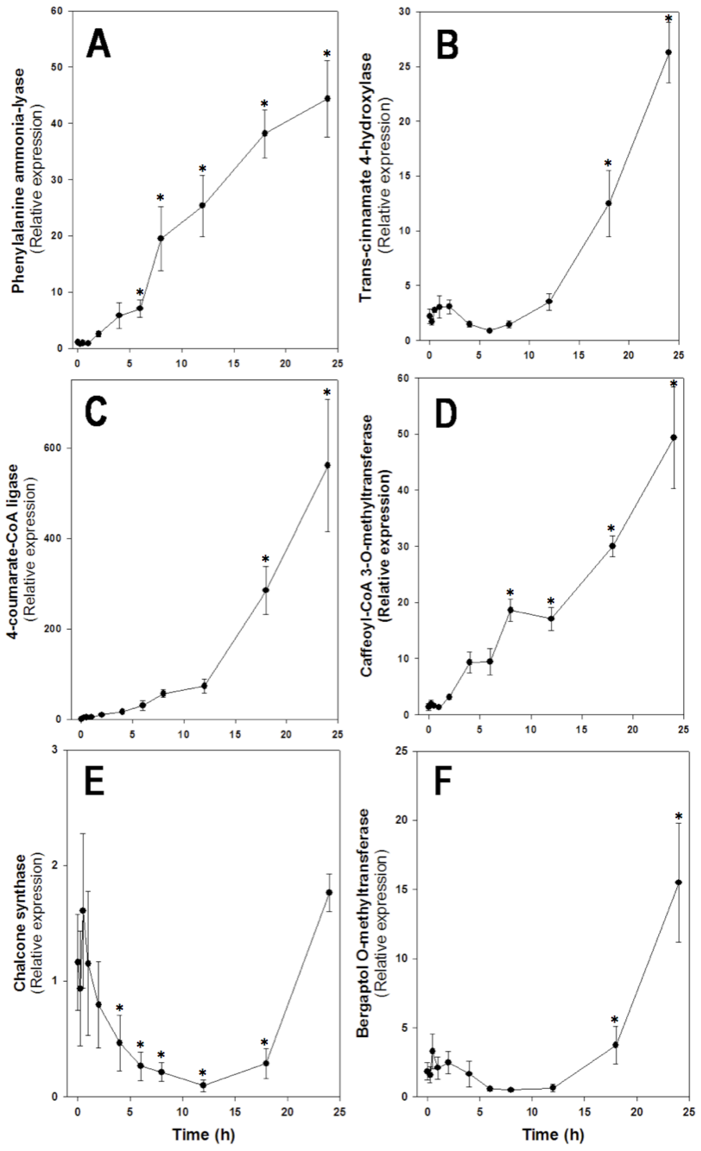

Fig. S6. Relative expression through time of genes related with the secondary metabolism in wounded carrots. Phenylalanine ammonia-lyase (PAL, A); Trans-cinnamate 4-hydroxylase (C4H, B); 4-coumarate-CoA ligase (4CL, C); Caffeoyl-CoA 3-O-methyltransferase (CCoAOMT, D); Bergaptol O-methyltransferase (BOMT, E). Data represents the mean of 4 replicates ± standard error of the mean. Data points with an asterisk (*) indicate statistical difference by the t-test ($p < 0.05$) with respect the relative expression of time 0 samples.

Table S5. Wound-induced subtractive cDNA library from carrots.

Sequence ID [i]	Size (bp)	Accession n° [ii]	E-value	Redundancy (from 979 secuences)	Gene Identification
**Stress signaling molecules**					
Ethylene biosynthesis genes					
SSH24-2-D6	699	IN PROCESS	1×10^{-99}	1	1-aminocyclopropane-1-carboxylate synthase [*Carica papaya*, AM113989]
SSH24-8-C2	1175	Already registered	0.0	9	S-adenosyl-L-methionine synthetase 1 [*Daucus carota*, AY583461]
SSH24-1-C7	540	IN PROCESS	9×10^{-157}	2	Methionine synthase [*Cicer arietinum*, EU924156]
SSH24-11-E6	152	IN PROCESS	4×10^{-53}	1	S-adenosylmethionine synthetase [*Petroselinum crispum*, M62757]
SSH24-2-H2	179	IN PROCESS	8×10^{-56}	1	S-adenosylmethionine synthetase [*Petroselinum crispum*, M62758]
SSH24-2-G8	606	IN PROCESS	0.0	4	S-adenosylhomocysteine hydrolase [*Petroselinum crispum*, M81885]
SSH24-3-A7	379	IN PROCESS	1×10^{-76}	1	Methylenetetrahydrofolate reductase, putative [*Vitis vinifera*, XM_002272694]
SSH24-9-G5	570	IN PROCESS	0.0	2	Methionine synthase, putative [*Vitis vinifera*, XM_002276402]
SSH24-2-B3	570	IN PROCESS	0.0	1	Cobalamine-independent methionine synthase [*Ricinus communis*, XM_002525663]
SSH24-1-G1	418	IN PROCESS	1×10^{-59}	2	Ethylene-responsive transcription factor 1B, putative [*Ricinus communis*, XM_002531493]
SSH24-1-D9	307	IN PROCESS	5×10^{-87}	3	Cobalamine-independent methionine synthase [*Coleus blumei*, Z49150]
Jasmonic acid biosynthesis genes					
SSH24-3-E8	233	IN PROCESS	1×10^{-43}	4	Microsomal omega-6 desaturase [*Glycine max*, AB188250]
SSH24-4-D1	595	IN PROCESS	0.0	3	Fatty acid desaturase/hydroxylase, putative [*Petroselinum crispum*, AF239834]
SSH24-6-H7	768	Already registered	0.0	3	Fatty acid desaturase/hydroxylase [*Daucus carota*, AF349965]
SSH24-7-G7	134	Already registered	8×10^{-48}	3	12-oxophytodienoate reductase, putative [*Daucus carota*, AJ297421]

Sequence ID [i]	Size (bp)	Accession n° [ii]	E-value	Redundancy (from 979 secuences)	Gene Identification
SSH24-3-D5	336	IN PROCESS	2×10^{-79}	4	3-hydroxyacyl-CoA dehyrogenase, putative [*Populus trichocarpa,* XM_002324390]
SSH24-1-G11	492	Already registered	0.0	7	Delta12-fatty acid acetylenase [*Daucus carota*, AY166774]
SSH24-5-G10	397	IN PROCESS	6×10^{-05}	1	Oleic acid desaturase [*Sesamum indicum,* AY770501]
SSH24-6-B6	223	IN PROCESS	1×10^{-36}	4	Microsomal omega-6-desaturase [*Glycine max*, AY954300]
SSH24-1-F12	374	IN PROCESS	1×10^{-58}	2	Lipoxygenase 5 [*Actinidia deliciosa*, DQ497796]
SSH24-5-E7	520	IN PROCESS	4×10^{-128}	2	Lipoxygenase 2 [*Actinidia deliciosa*, DQ497797]
SSH24-10-F8	159	IN PROCESS	4×10^{-21}	1	Delta-12 desaturase [*Lepidium virginicum,* DQ518315]
SSH24-9-E4	116	IN PROCESS	8×10^{-15}	1	Delta-12 desaturase [*Lepidium virginicum,* DQ518318]
SSH24-5-A7	651	IN PROCESS	1×10^{-74}	1	Omega-6 fatty acid desaturase 2 [*Portulaca oleracea*, FJ472350]
SSH24-6-E9	383	IN PROCESS	2×10^{-157}	1	Omega-6 fatty acid desaturase [*Petroselinum crispum*, U86072]
SSH24-4-B7	651	IN PROCESS	2×10^{-75}	3	D12 oleate desaturase [*Solanum commersonii*, X92847]
SSH24-6-D1	252	IN PROCESS	5×10^{-29}	1	Delta12 fatty acid desaturase, putative [*Vitis vinifera*, XM_002279203]
SSH24-1-H7	495	IN PROCESS	4×10^{-116}	8	12-oxophytodienoate reductase, putative [*Vitis vinifera*, XM_002281083]
SSH24-5-B6	971	IN PROCESS	0.0	3	3-ketoacyl-CoA thiolase, putative [*Populus trichocarpa,* XM_002301534]
SSH24-3-G5	504	IN PROCESS	5×10^{-83}	1	Delta12 fatty acid desaturase, putative [*Populus trichocarpa*, XM_002323248]
SSH24-3-B11	449	IN PROCESS	3×10^{-104}	3	12-oxophytodienoic acid 10,10-reductase, putative [*Populus trichocarpa*, XM_002327253]
SSH24-9-E5	329	IN PROCESS	1×10^{-64}	1	12-oxophytodienoate reductase, putative [*Populus trichocarpa*, XM_002336702]
SSH24-7-A1	288	IN PROCESS	8×10^{-65}	6	3-ketoacyl-CoA thiolase B, putative [*Ricinus communis*, XM_002521077]
SSH24-6-F4	897	IN PROCESS	0.0	1	3-ketoacyl-CoA thiolase B [*Mangifera indica*, XR_077410]
SSH24-10-C7	265	IN PROCESS	6×10^{-173}	1	3-ketoacyl-CoA thiolase [*Populus trichocarpa*, XM_002299248]

Sequence ID [i]	Size (bp)	Accession n° [ii]	E-value	Redundancy (from 979 secuences)	Gene Identification
SSH24-8-B9	905	IN PROCESS	1×10^{-11}	1	2,4-dienoyl-CoA reductase, putative [*Ricinus communis*, XM_002517269]
SSH24-9-F8	397	IN PROCESS	8×10^{-04}	1	Microsomal oleic acid desaturase [*Sesamum indicum*, AY770501]
Reactive oxygen species biosynthesis and modulation/detoxification genes					
SSH24-1-D7	555	IN PROCESS	6×10^{-146}	20	NADH-ubiquinone oxidoreductase, putative [*Prunus armeniaca*, AF139496]
SSH24-2-D7	561	IN PROCESS	6×10^{-140}	9	Glutathione S-transferase [*Petroselinum crispum*, AF177944]
SSH24-1-B1	912	IN PROCESS	2×10^{-85}	14	NADPH:quinone reductase [*Populus trichocarpa*, EF144480]
SSH24-7-D2	161	IN PROCESS	9×10^{-42}	1	Glutathione peroxidase [*Litchi chinensis*, FJ172343]
SSH24-8-F5	548	IN PROCESS	1×10^{-177}	2	NADPH:quinone reductase, putative [*Vitis vinifera*, XM_002267141]
SSH24-11-C4	631	IN PROCESS	3×10^{-69}	1	Glutathione S-transferase, putative [*Vitis vinifera*, XM_002275846]
SSH24-6-D3	286	IN PROCESS	8×10^{-59}	2	Thioredoxin H-type 1, putative [*Vitis vinifera*, XM_002280537]
SSH24-6-C9	265	IN PROCESS	1×10^{-61}	3	Glycolate oxidase, putative [*Vitis vinifera*, XM_002278068]
SSH24-4-E6	531	IN PROCESS	3×10^{-105}	1	Peroxiredoxin, putative [*Vitis vinifera*, XM_002283616]
SSH24-6-G9	223	IN PROCESS	5×10^{-47}	5	Peroxidase 12 precursor, putative [*Ricinus communis*, XM_002524270]
SSH24-10-E5	509	IN PROCESS	1×10^{-72}	25	Glutathione-s-transferase omega, putative [*Ricinus communis*, XM_002525158]
SSH24-10-H9	299	IN PROCESS	7×10^{-66}	1	NADH-ubiquinone oxidoreductase, putative [*Ricinus communis*, XM_002531885]
SSH24-5-E8	653	IN PROCESS	0.0	4	Anionic peroxidase [*Petroselinum crispum*, L36981]
SSH24-10-H2	595	IN PROCESS	9×10^{-94}	1	Laccase, putative [*Vitis vinifera*, XM_002272582]
SSH24-6-G8	337	IN PROCESS	7×10^{-45}	1	Anionic peroxidase precursor, putative [*Ricinus communis,* XM_002521822]
SSH24-2-A6	559	IN PROCESS	2×10^{-102}	6	Peroxidase [*Panax ginseng*, AB232686]
SSH24-7-A10	403	IN PROCESS	1×10^{-26}	2	Glutathione S-transferase, putative [*Vitis vinifera,* XM_002273835]

Sequence ID (i)	Size (bp)	Accession n° (ii)	E-value	Redundancy (from 979 secuences)	Gene Identification
SSH24-8-E1	237	IN PROCESS	2×10^{-14}	2	Glutathione s-transferase, putative [*Ricinus communis,* XM_002523950]
Genes involved in signaling the NADPH oxidase production of reactive oxygen species					
SSH24-7-A11	731	IN PROCESS	0.0	3	ATP-binding cassette transporter, putative [*Vitis vinifera*, XR_078070]
SSH24-2-B10	347	IN PROCESS	2×10^{-05}	1	ATP-binding cassette transporter, putative [*Ricinus communis,* XM_002527284]
SSH24-8-H9	216	IN PROCESS	1×10^{-42}	1	ATP-binding cassette transporter, putative [*Populus trichocarpa*, XM_002304676]
SSH24-10-G1	923	Already registered	0.0	41	Exo-polygalacturonase [*Daucus carota*, D29664]
SSH24-6-F11	444	IN PROCESS	3×10^{-14}	1	Calcium-binding protein [*Olea europaea*, AF078680]
SSH24-7-C5	534	IN PROCESS	2×10^{-12}	1	Calcium-binding protein, putative [*Populus trichocarpa*, XM_002312286]
**Carbon source**					
Sugars transport genes					
SSH24-1-E12	330	IN PROCESS	1×10^{-81}	3	Hexose transporte-like protein [*Apium graveolens*, AF393808]
SSH24-3-F2	340	IN PROCESS	6×10^{-61}	2	Sugar transporter, putative [*Ricinus communis,* XM_002510945]
SSH24-2-D1	167	IN PROCESS	6×10^{-19}	1	Sugar transporter, putative [*Ricinus communis*, XM_002517170]
Glycolisis and pentose phosphate pathway genes					
SSH24-9-F3	354	IN PROCESS	1×10^{-153}	1	Cofactor-independent phosphoglyceromutase [*Apium graveolens*, AJ132256]
SSH24-8-G9	518	IN PROCESS	3×10^{-169}	3	6-phosphogluconate dehydrogenase, putative [*Vitis vinifera,* XM_002270948]
SSH24-9-H6	191	IN PROCESS	2×10^{-51}	1	Fructose-1,6-bisphosphate aldolase, putative [*Vitis vinifera,* XM_002283345]
SSH24-10-E8	518	IN PROCESS	8×10^{-170}	2	6-phosphogluconate dehydrogenase, putative [*Populus trichocarpa,* XM_002311387.1]
SSH24-8-A11	269	IN PROCESS	6×10^{-66}	1	Fructose-1,6-bisphosphate aldolase, putative [*Vitis vinifera,* XM_002283345]

Sequence ID [(i)]	Size (bp)	Accession n° [(ii)]	E-value	Redundancy (from 979 secuences)	Gene Identification
Shikimate pathway genes					
SSH24-1-F9	927	IN PROCESS	0.0	8	3-deoxy-D-arabino-heptulosonate [*Petroselinum crispum*, AF012864]
SSH24-10-E2	733	IN PROCESS	0.0	3	5-enolpyruvylshikimate 3-phosphate synthase [*Camptotheca acuminata*, AY639815]
Aromatic amino acid biosynthesis genes					
SSH24-4-D12	101	IN PROCESS	2×10^{-15}	1	Chorismate mutase, putative [*Vitis vinifera*, XM_002284083]
SSH24-3-C5	459	IN PROCESS	2×10^{-82}	2	Chorismate mutase-prephenate dehydratase, putative [*Vitis vinifera*, XM_002268665]
SSH24-9-D7	148	IN PROCESS	5×10^{-32}	3	Prephenate dehydratase, putative [*Ricinus communis*, XM_002527418]
**Secondary metabolites biosynthesis genes**					
Phenolic metabolism genes *Hydroxycinnamic acids biosynthesis genes*					
SSH24-3-A9	333	IN PROCESS	3×10^{-128}	11	Cinnamate 4-hydroxylase [*Ammi majus*, AY219918]
SSH24-11-C9	866	Already registered	0.0	17	Phenylalanine ammonia-lyase (gDcPAL1) [*Daucus carota*, D85850]
SSH24-5-D9	392	IN PROCESS	1×10^{-153}	7	Trans-cinnamate 4-monooxygenase [*Petroselinum crispum*, L38898]
SSH24-2-G6	495	IN PROCESS	1×10^{-172}	2	4-coumarate-coa ligase [*Petroselinum crispum*, X13325]
SSH24-4-E3	289	IN PROCESS	5×10^{-30}	10	4-coumarate-coa ligase [*Vitis vinifera*, XM_002270324]
SSH24-7-H10	733	IN PROCESS	6×10^{-97}	9	4-coumarate-coa ligase [*Populus trichocarpa*, XM_002304328]
Flavonoids biosynthesis genes					
SSH24-7-E10	432	IN PROCESS	3×10^{-160}	1	Glycosyltransferase, putative [*Lycium barbarum*, AB360610]
SSH24-5-F9	936	Already registered	2×10^{-104}	3	Chalcone synthase 2 [*Daucus carota*, AJ006780]
SSH24-5-G11	813	IN PROCESS	9×10^{-58}	3	Tetrahydroxychalcone 2'-glucosyltransferase [*Catharanthus roseus*, AB294401]
SSH24-6-D5	364	IN PROCESS	7×10^{-48}	1	Hydroxycinnamoyl-CoA:anthocyanin 3-O-glucoside-6"-O-acyltransferase [*Petunia x hybrida*, AB026495]
SSH24-8-E6	432	Already registered	0.0	1	Favone synthase I [*Daucus carota*, AY817675]

Sequence ID (i)	Size (bp)	Accession n° (ii)	E-value	Redundancy (from 979 secuences)	Gene Identification
Lignin biosynthesis genes					
SSH24-7-F2	602	IN PROCESS	6×10^{-155}	4	Cinnamoyl-CoA reductase [*Codonopsis lanceolata*, AB243011]
SSH24-7-G3	196	IN PROCESS	4×10^{-28}	1	NADPH-dependent cinnamyl alcohol dehydrogenase [*Quercus suber*, AY362455]
SSH24-6-F12	516	IN PROCESS	0.0	1	Caffeoyl-CoA O-methyltransferase [*Ammi majus*, AY620245]
SSH24-5-F5	755	IN PROCESS	0.0	1	Cinnamyl alcohol dehydrogenase [*Aralia cordata*, D13991]
SSH24-1-C12	465	IN PROCESS	2×10^{-112}	1	Cinnamoyl-CoA reductase-like [*Panax ginseng*, FJ002428]
SSH24-4-G11	138	IN PROCESS	1×10^{-25}	1	Cinnamoyl CoA reductase [*Betula luminifera*, FJ410450]
SSH24-9-D9	513	IN PROCESS	0.0	2	Caffeoyl-CoA 3-O-methyltransferase [*Petroselinum crispum,* M69184]
SSH24-11-D2	215	IN PROCESS	6×10^{-27}	2	Cinnamoyl-CoA reductase [*Populus trichocarpa*, XM_002332042]
Lignans biosynthesis genes					
SSH24-8-H7	813	IN PROCESS	1×10^{-175}	9	Isoflavone reductase-like protein 5 [*Vitis vinifera*, BN000710]
Furanocoumarin biosynthesis genes					
SSH24-10-D12	393	IN PROCESS	5×10^{-144}	30	Bergaptol O-methyltransferase [*Glehnia littoralis*, AB363638]
Volatile benzenoid ester biosynthesis genes					
SSH24-7-G9	479	IN PROCESS	1×10^{-49}	7	Benzoyl coenzyme A: benzyl alcohol benzoyl transferase [*Nicotiana tabacum,* AF500202]
SSH24-2-B12	577	IN PROCESS	6×10^{-14}	1	Benzoyl coenzyme A: benzyl alcohol benzoyl transferase [*Petunia x hybrida*, AY563157]
Cardenolide pathway genes					
SSH24-5-B4	298	IN PROCESS	4×10^{-56}	2	Progesterone 5-beta-reductase [*Digitalis lutea*, DQ213021]
Alkaloids biosynthesis genes					
SSH24-4-D5	264	IN PROCESS	2×10^{-10}	9	Berberine bridge, putative [*Panax ginseng*, DQ384527]
SSH24-6-A11	188	IN PROCESS	1×10^{-21}	1	Berberine bridge, putative [*Arabidopsis thaliana*, NM_100078.2]
SSH24-6-C8	326	IN PROCESS	1×10^{-12}	2	Berberine bridge, putative [*Vitis vinifera*, XM_002268325]
SSH24-3-D12	306	IN PROCESS	1×10^{-05}	4	Berberine bridge, putative [*Vitis vinifera*, XM_002268570]

Sequence ID (i)	Size (bp)	Accession n° (ii)	E-value	Redundancy (from 979 secuences)	Gene Identification
SSH24-3-C8	336	IN PROCESS	2×10^{-41}	3	Reticuline oxidase precursor, putative [*Vitis vinifera*, XM_002266993]
SSH24-4-E7	525	IN PROCESS	8×10^{-75}	6	Reticuline oxidase precursor, putative [*Vitis vinifera*, XM_002269426]
SSH24-7-H8	711	IN PROCESS	6×10^{-116}	3	Reticuline oxidase precursor, putative [*Populus trichocarpa*, XM_002333922]
SSH24-3-D4	187	IN PROCESS	3×10^{-17}	3	Reticuline oxidase precursor, putative [*Ricinus communis*, XM_002523110]
SSH24-10-F2	369	IN PROCESS	3×10^{-71}	1	Reticuline oxidase precursor, putative [*Ricinus communis*, XM_002523118]
SSH24-3-C11	524	IN PROCESS	2×10^{-82}	1	Reticuline oxidase precursor, putative [*Ricinus communis,* XM_002538779]
SSH24-9-F9	427	IN PROCESS	2×10^{-42}	1	Reticuline oxidase precursor, putative [*Ricinus communis,* XM_002523116]
SSH24-5-F3	962	IN PROCESS	2×10^{-10}	1	Polyneuridine-aldehyde esterase precursor, putative [*Ricinus communis*, XM_002522306]
SSH24-4-G1	375	IN PROCESS	3×10^{-08}	1	S-norcoclaurine synthase 2 [*Papaver somniferum*, AY860501]
Phenylpropene biosynthesis genes					
SSH24-5-G12	404	IN PROCESS	3×10^{-59}	1	SAM:t-anol/isoeugenol O-methyltransferase [*Pimpinella anisum,* EU925389]
Terpenoids biosynthesis genes					
SSH24-10-C5	144	IN PROCESS	6×10^{-23}	1	1-deoxy-D-xylulose 5-phosphate reductoisomerase [*Arabidopsis thaliana*, NM_125674.2]
Phytosterols biosynthesis genes					
SSH24-2-A2	427	IN PROCESS	7×10^{-87}	1	Sterol 22-desaturase, similar [*Vitis vinifera*, XM_002276577]
SSH24-10-C11	312	IN PROCESS	2×10^{-40}	3	Sterol desaturase, putative [*Ricinus communis*, XM_002526466]
SSH24-10-C2	310	IN PROCESS	4×10^{-37}	1	Sterol desaturase, putative [*Ricinus communis*, XM_002526465]
**Other disease and stress response genes**					
SSH24-5-C7	689	IN PROCESS	1×10^{-74}	1	Alcohol dehydrogenase, putative [*Ricinus communis*, XP_002525379]

Sequence ID [i]	Size (bp)	Accession n° [ii]	E-value	Redundancy (from 979 secuences)	Gene Identification
SSH24-7-G10	269	Already registered	3×10^{-134}	2	Dcarg-1 [*Daucus carota,* AB027501]
SSH24-9-G4	794	Already registered	0.0	16	Pathogenesis-related protein-like protein 1 [*Daucus carota,* AB082377]
SSH24-1-A11	207	IN PROCESS	2×10^{-14}	12	Protease inhibitor 2 [*Zinnia elegans*, AB091074]
SSH24-9-G1	544	Already registered	0.0	4	Short chain alcohol dehydrogenase-like [*Daucus carota,* AB104856]
SSH24-2-F2	389	IN PROCESS	1×10^{-12}	1	[*Populus trichocarpa*, XM_002303561]
SSH24-9-A9	386	IN PROCESS	4×10^{-51}	1	Chitinase III [*Vitis vinifera*, AB105374]
SSH24-8-F4	593	Already registered	0.0	41	CAPSE mRNA for cell attachment protein in somatic embryogenesis [*Daucus carota,* AB104856]
SSH24-8-E9	341	Already registered	7×10^{-168}	9	Pathogenesis-related protein-like protein 1 [*Daucus carota*, AB127984]
SSH24-11-A11	598	IN PROCESS	0.0	3	Chitinase precursor [*Petroselinum crispum,* AF141374]
SSH24-5-C6	276	IN PROCESS	2×10^{-10}	1	Synthetic construct major pollen allergen Artv1 [Synthetic construct, AF502559]
SSH24-5-E4	343	IN PROCESS	9×10^{-34}	2	Polygalacturonase-inhibiting protein, putative [*Rubus idaeus*, AJ620354]
SSH24-10-A10	699	IN PROCESS	2×10^{-40}	2	Alcohol dehydrogenase [*Solanum tuberosum*, X92179]
SSH24-4-E12	505	IN PROCESS	8×10^{-18}	1	Polyphenol oxidase [*Vitis vinifera*, XM_002276076]
SSH24-1-A3-1	288	IN PROCESS	1×10^{-24}	2	Monooxygenase, putative [*Solanum tuberosum,* AB061259]
SSH24-4-H3	421	IN PROCESS	4×10^{-45}	2	Heterogeneous nuclear ribonucleoprotein 27C [*Ricinus communis*, XM_002528331]
SSH24-1-B10	694	Already registered	0.0	31	Extracellular dermal glycoprotein [*Daucus carota*, D14550]
SSH24-11-D1	269	IN PROCESS	2×10^{-53}	1	Polyphenol oxidase [*Camellia nitidissima*, FJ597757]
SSH24-10-G4	217	IN PROCESS	7×10^{-39}	8	2-alkenal reductase [*Artemisia annua*, FJ750460]
SSH24-11-E2	848	IN PROCESS	6×10^{-111}	6	Polyphenol oxidase [*Taraxacum officinale*, FM178478]
SSH24-9-F10	668	Already registered	0.0	7	Secreted protein (EP4b) [*Daucus carota*, L36957]

Sequence ID [(i)]	Size (bp)	Accession n° [(ii)]	E-value	Redundancy (from 979 secuences)	Gene Identification
SSH24-4-F6	246	Already registered	2×10^{-96}	1	Class IV chitinase EP3-3/E7 (EP3) [*Daucus carota*, U52847]
SSH24-1-A1	343	IN PROCESS	2×10^{-112}	3	Pathogenesis-related protein type B [*Petroselinum crispum*, X12573]
SSH24-9-G9	535	IN PROCESS	6×10^{-165}	4	Pathogenesis-related protein type A [*Petroselinum crispum*, X12574]
SSH24-2-C11	716	IN PROCESS	0.0	3	Pathogenesis-related protein 2 [*Petroselinum crispum*, X58698]
SSH24-4-E2	335	IN PROCESS	1×10^{-32}	1	Chitinase, putative [*Vitis vinifera*, XM_002270543]
SSH24-8-G8	174	IN PROCESS	2×10^{-31}	1	Alcohol dehydrogenase, putative [*Vitis vinifera*, XM_002279390]
SSH24-9-C6	584	IN PROCESS	3×10^{-144}	2	Heat shock 70 kDa protein, putative [*Ricinus communis*, XM_002528153]
SSH24-4-F3	277	IN PROCESS	2×10^{-48}	1	Pectinesterase, putative [*Vitis vinifera*, XM_002280410]
SSH24-4-E4	711	IN PROCESS	8×10^{-140}	1	Pectinesterase, putative [*Vitis vinifera*, XM_002282242]
SSH24-7-E4	675	IN PROCESS	2×10^{-65}	1	Heat shock transcription factor, putative [*Vitis vinifera*, XM_002284180]
SSH24-9-C4	545	IN PROCESS	4×10^{-79}	3	MtN19-like protein [*Vitis vinifera*, XM_002284703]
SSH24-11-G4	634	IN PROCESS	5×10^{-180}	2	Sulfite reductase, putative [*Vitis vinifera*, XM_002285362]
SSH24-5-H12	806	IN PROCESS	2×10^{-63}	1	Heat shock transcription factor, putative [*Populus trichocarpa*, XM_002307987]
SSH24-11-F8	257	IN PROCESS	6×10^{-41}	1	Glucan endo-1,3-beta-glucosidase precursor, putative [*Populus trichocarpa*, XM_002312061]
SSH24-2-E8	564	IN PROCESS	6×10^{-153}	1	Pectinesterase-2 precursor, putative [*Populus trichocarpa*, XM_002322365]
SSH24-10-E11	510	IN PROCESS	4×10^{-47}	6	Polygalacturonase inhibitor [*Actinidia deliciosa*, Z49063]
SSH24-9-D4	281	IN PROCESS	1×10^{-31}	12	Mutt/nudix hydrolase, putative [*Ricinus communis*, XM_002524337]
SSH24-1-B9	332	IN PROCESS	1×10^{-38}	3	Hevamine-A precursor, putative [*Ricinus communis*, XM_002513566]
SSH24-5-C5	361	IN PROCESS	6×10^{-24}	4	Potato inhibitor I, putative [*Populus trichocarpa*, XM_002334086]
SSH24-5-D11	340	IN PROCESS	4×10^{-76}	10	2-alkenal reductase, putative [*Populus trichocarpa*, XM_002331617]

Sequence ID (i)	Size (bp)	Accession n° (ii)	E-value	Redundancy (from 979 secuences)	Gene Identification
SSH24-1-E4	542	IN PROCESS	6×10^{-83}	1	Heat shock factor, putative [*Populus trichocarpa*, XM_002323284]
SSH24-9-B10	437	IN PROCESS	2×10^{-47}	3	Zn-dependent alcohol dehydrogenases, putative [*Populus trichocarpa*, XM_002324658]
SSH24-4-C2	600	IN PROCESS	0.0	1	Major allergen Dau c 1/1 [*Daucus carota*, Z84376]
SSH24-10-D2	272	IN PROCESS	2×10^{-02}	3	Alcohol dehydrogenase [*Vitis vinifera*, XM_002281229]
SSH24-10-H8	299	IN PROCESS	2×10^{-21}	1	Protease inhibitor, putative [*Populus trichocarpa*, XM_002334086]
SSH24-1-D6	301	IN PROCESS	4×10^{-12}	5	Miraculin homologue, putative [*Youngia japonica*, AB023648]
SSH24-2-H10	320	IN PROCESS	5×10^{-11}	2	Patatin-like protein 3 [*Nicotiana tabacum*, AF158253]
SSH24-2-A12	501	IN PROCESS	1×10^{-02}	2	Kunitz trypsin inhibitor [*Nicotiana tabacum*, FJ494920]
SSH24-9-E12	415	IN PROCESS	2×10^{-17}	1	Light-inducible protein atls1, putative [*Ricinus communis*, XM_002526640]
SSH24-5-B12	826	IN PROCESS	6×10^{-180}	1	Lysine histidine transporter 1, putative [*Vitis vinifera*, XM_002265272]
SSH24-5-H8	633	Already registered	1×10^{-36}	1	P40-like protein [*Daucus carota*, AB012702]
SSH24-8-C4	359	Already registered	1×10^{-178}	1	NADP specific isocitrate dehydrogenase [*Daucus carota*, AB019327]
SSH24-5-F2	505	IN PROCESS	2×10^{-125}	1	Nucleoside diphosphate kinase 1 [*Codonopsis lanceolata*, AB126060]
SSH24-9-H1	269	IN PROCESS	1×10^{-44}	2	WAKL1 gene [*Nicotiana tabacum*, AB291545]
SSH24-2-H12	241	IN PROCESS	1×10^{-67}	1	Serine hydroxymethyltransferase 1 [*Glycine max*, EU912419]
SSH24-7-B6	557	IN PROCESS	5×10^{-109}	1	RNA binding protein, putative [*Nicotiana tabacum*, AF029351]
SSH24-7-G6	488	IN PROCESS	0.0	1	Acyl carrier protein [*Daucus carota*, AF083950]
SSH24-7-G8	524	Already registered	0.0	2	Poly(A)-binding protein [*Daucus carota*, AF349964.1
SSH24-4-C5	505	IN PROCESS	5×10^{-77}	1	Adenosine 5'-phosphosulfate reductase [*Glycine max*, AF452450]
SSH24-3-C2	102	IN PROCESS	2×10^{-16}	1	Monooxygenase, putative [*Arabidopsis thaliana*, AK227657]

Sequence ID [i]	Size (bp)	Accession n° [ii]	E-value	Redundancy (from 979 secuences)	Gene Identification
SSH24-3-F7	202	IN PROCESS	6×10^{-14}	3	Glutathione-s-transferase omega, putative [*Ricinus communis*, XM_002525158]
SSH24-1-C11	550	IN PROCESS	5×10^{-103}	1	Cysteine hydrolase, putative [*Vitis vinifera*, XM_002270860]
SSH24-3-F11	329	IN PROCESS	3×10^{-78}	1	Serine/threonine protein kinase, putative [*Ricinus communis*, XM_002523507]
SSH24-6-G2	211	IN PROCESS	2×10^{-59}	3	Serine/threonine protein phosphatase, putative [*Arabidopsis thaliana*, NM_103920.3]
SSH24-8-B7	576	IN PROCESS	1×10^{-41}	1	Glucose-methanol-choline oxidoreductase, putative [*Ricinus communis*, XM_002527358]
SSH24-8-D9	275	IN PROCESS	2×10^{-34}	1	Membrane transport protein, putative [*Populus trichocarpa*, XM_002301858]
SSH24-1-H8	240	IN PROCESS	6×10^{-34}	1	Ribosome-associated protein p40-like [*Solanum tuberosum*, DQ207864]
SSH24-9-C12	530	IN PROCESS	1×10^{-97}	3	Peroxisomal membrane protein 2, pxmp2, putative [*Ricinus communis*, XM_002518406]
SSH24-1-A3	168	IN PROCESS	7×10^{-25}	2	60S ribosomal protein L21-like protein [*Solanum tuberosum*, DQ191668]
SSH24-11-H10	272	IN PROCESS	1×10^{-37}	1	Protein phosphatase 2c, putative [*Ricinus communis*, XM_002534061]
SSH24-11-G5	472	IN PROCESS	1×10^{-57}	1	S-locus-specific glycoprotein precursor, putative [*Ricinus communis*, XM_002525833]
SSH24-2-B5	604	IN PROCESS	2×10^{-39}	1	Kinase, putative [*Vitis vinifera*, XM_002277295]
SSH24-7-B4	289	IN PROCESS	6×10^{-54}	1	UDP-glucose 4-epimerase [*Solanum lycopersicum,* AY197749]
SSH24-3-F8	286	IN PROCESS	1×10^{-125}	1	Variable lymphocyte receptor (VLR) [*Petromyzon marinus*, AY577941]
SSH24-10-F7	473	Already registered	0.0	1	Ribosomal protein S7 (rps7) and cytochrome oxidase subunit 1-2 [*Daucus carota*, AY820131]
SSH24-10-B10	221	IN PROCESS	1×10^{-55}	1	40S ribosomal protein S3, putative [*Ricinus communis*, XM_002514015]
SSH24-2-H6	287	IN PROCESS	2×10^{-73}	1	GTP cyclohydrolase II/3,4-dihydroxy-2-butanone 4-phosphate synthase [*Malus x domestica*, AF403706]
SSH24-4-E9	280	IN PROCESS	3×10^{-71}	2	Ribosomal_L7Ae protein, putative [*Vitis vinifera*, XM_002281001]

Sequence ID [i]	Size (bp)	Accession n° [ii]	E-value	Redundancy (from 979 secuences)	Gene Identification
SSH24-2-E3	447	IN PROCESS	3×10^{-41}	1	Amino acid transporter, putative [*Ricinus communis*, XM_002528370]
SSH24-7-C8	198	IN PROCESS	6×10^{-36}	1	60S ribosomal protein L13a, putative [*Zea mays*, NM_001137336]
SSH24-2-E2	326	IN PROCESS	1×10^{-18}	1	Cytochrome P450, putative [*Glycine max*, Y10493]
SSH24-11-F12	983	Already registered	0.0	1	Elongation factor 1-alpha [*Daucus carota*, D12709]
SSH24-3-B5	515	IN PROCESS	5×10^{-160}	1	Aconitase [*Cucurbita cv. Kurokawa Amakuri*, D29629]
SSH24-9-F6	709	IN PROCESS	2×10^{-84}	1	Cytochrome P450 monooxygenase [*Petunia x hybrida*, DQ099541]
SSH24-4-B5	419	IN PROCESS	6×10^{-75}	1	60S ribosomal protein, putative [*Solanum tuberosum*, DQ294270]
SSH24-3-F9	148	IN PROCESS	6×10^{-69}	1	26S ribosomal RNA [*Panax ginseng*, GQ178085]
SSH24-7-F8	446	IN PROCESS	1×10^{-109}	1	60S ribosomal protein L13A [*Arabidopsis thaliana*, NM_111591]
SSH24-4-D6	613	IN PROCESS	5×10^{-40}	1	Epoxide hydrolase [*Solanum tuberosum*, U02494]
SSH24-6-E10	172	IN PROCESS	3×10^{-54}	1	Ribosomal protein, putative [*Daucus carota*, U47095]
SSH24-4-H1	787	IN PROCESS	2×10^{-167}	1	60S ribosomal protein [*Vitis vinifera*, XM_002262661]
SSH24-6-E7	480	IN PROCESS	2×10^{-37}	1	Patatin, putative [*Vitis vinifera*, XM_002262906]
SSH24-9-B8	294	IN PROCESS	2×10^{-34}	1	AMP dependent ligase, putative [*Vitis vinifera*, XM_002263765]
SSH24-1-B8	240	IN PROCESS	4×10^{-30}	1	AMP dependent ligase, putative [*Ricinus communis*, XM_002521697]
SSH24-2-G9	547	IN PROCESS	1×10^{-141}	1	Inosine-5'-monophosphate dehydrogenase, putative [*Vitis vinifera*, XM_002265010]
SSH24-10-G12	440	IN PROCESS	2×10^{-110}	1	Stearoyl-acyl-carrier protein desaturase, putative [*Vitis vinifera*, XM_002265150]
SSH24-6-C7	782	IN PROCESS	8×10^{-128}	1	Lysine histidine transporter 1, putative [*Vitis vinifera*, XM_002265272]
SSH24-9-E3	406	IN PROCESS	2×10^{-30}	2	Fatty acid hydroxylase, putative [*Vitis vinifera*, XM_002265649]
SSH24-7-E6	547	IN PROCESS	3×10^{-74}	6	Aldo/keto reductase, putative [*Vitis vinifera*, XM_002265891]
SSH24-2-D9	379	IN PROCESS	1×10^{-89}	1	Aldo/keto reductase, putative [*Vitis vinifera*, XM_002266241]
SSH24-10-D1	538	IN PROCESS	3×10^{-24}	1	Zinc finger protein, putative [*Ricinus communis*, XM_002522244]

Sequence ID (i)	Size (bp)	Accession n° (ii)	E-value	Redundancy (from 979 secuences)	Gene Identification
SSH24-5-E3	521	IN PROCESS	1×10^{-16}	1	Zinc finger protein, putative [*Ricinus communis*, XM_002533195]
SSH24-4-C3	483	IN PROCESS	2×10^{-144}	1	Caseinolytic protease, putative [*Vitis vinifera,* XM_002266344]
SSH24-4-D2	450	IN PROCESS	8×10^{-155}	1	60S ribosomal protein L13a protein [*Vitis vinifera,* XM_002267455]
SSH24-2-G11	453	IN PROCESS	5×10^{-70}	1	Serine/threonine protein kinase, putative [*Vitis* vinifera, XM_002267636]
SSH24-9-D6	262	IN PROCESS	1×10^{-62}	1	Radical sam protein, putative [*Vitis vinifera,* XM_002268256]
SSH24-8-F10	269	IN PROCESS	1×10^{-44}	1	Ankyrin repeat-containing protein, putative [*Vitis vinifera*, XM_002268406]
SSH24-7-H2	235	IN PROCESS	1×10^{-61}	1	UDP-glucose 4-epimerase, putative [*Vitis vinifera,* XM_002268743]
SSH24-9-D5	734	IN PROCESS	1×10^{-163}	1	Ubiquinone/menaquinone biosynthesis methyltransferase, putative [*Vitis vinifera*, XM_002271722]
SSH24-9-H7	289	IN PROCESS	2×10^{-47}	3	Lipase like [*Vitis vinifera,* XM_002271766]
SSH24-8-B11	442	IN PROCESS	3×10^{-129}	1	Adenosine kinase [*Vitis vinifera*, XM_002272301]
SSH24-5-D5	522	IN PROCESS	2×10^{-43}	1	Monoxygenase, putative [Ricinus communis, XM_002530028]
SSH24-3-B6	307	IN PROCESS	5×10^{-74}	1	Cwf15/Cwc15 cell cycle control protein [*Vitis vinifera,* XM_002272417]
SSH24-5-D1	326	IN PROCESS	2×10^{-42}	1	Pollen allerg 1 [*Vitis vinifera,* XM_002273860]
SSH24-2-F5	521	IN PROCESS	2×10^{-37}	1	Short-chain dehydrogenase, putative [*Vitis vinifera,* XM_002274896]
SSH24-10-E4	384	IN PROCESS	1×10^{-90}	1	Transcription factor IWS1, putative [*Vitis vinifera*, XM_002276847]
SSH24-4-C10	153	IN PROCESS	1×10^{-21}	1	Reverse transcriptases, putative [*Vitis vinifera*, XM_002277654]
SSH24-2-F9	389	IN PROCESS	8×10^{-54}	1	Major facilitator, putative [*Vitis vinifera,* XM_002277715]
SSH24-11-C11	430	IN PROCESS	2×10^{-99}	1	Glutathione S-transferas [*Vitis vinifera,* XM_002277850]
SSH24-4-H10	427	IN PROCESS	3×10^{-92}	1	Glycine cleavage system h protein, putative [*Ricinus communis*, XM_002528116]
SSH24-2-C5	498	IN PROCESS	1×10^{-96}	1	Peroxisomal membrane protein, putative [*Vitis vinifera,* XM_002279452]

Sequence ID (i)	Size (bp)	Accession n° (ii)	E-value	Redundancy (from 979 secuences)	Gene Identification
SSH24-9-H2	387	IN PROCESS	2×10^{-55}	1	MATE efflux protein family, putative [*Vitis vinifera*, XM_002280387]
SSH24-3-F6	605	IN PROCESS	5×10^{-21}	1	MATE efflux protein family, putative [*Vitis vinifera*, XM_002265897]
SSH24-7-E12	521	IN PROCESS	5×10^{-122}	2	Nuclear acid binding protein, putative [*Vitis vinifera*, XM_002280565]
SSH24-4-F1	288	IN PROCESS	1×10^{-50}	1	Serine/Threonine protein kinases, catalytic domain [*Vitis vinifera*, XM_002282409]
SSH24-3-B4	182	IN PROCESS	7×10^{-38}	1	Proteinase kinase, putative [*Vitis vinifera*, XM_002282851]
SSH24-8-H12	615	IN PROCESS	1×10^{-98}	1	Type 1 glutamine amidotransferase (GATase1)-like domain [*Vitis vinifera*, XM_002283027]
SSH24-4-C11	440	IN PROCESS	7×10^{-56}	1	Glycosyltransferase, putative [*Vitis vinifera*, XM_002284345]
SSH24-6-C1	300	IN PROCESS	5×10^{-43}	2	2-polyprenyl-6-methoxyphenol hydroxylase, putative [*Vitis vinifera*, XM_002284701]
SSH24-1-E6	694	IN PROCESS	2×10^{-108}	1	NADH pyrophosphatase, putative [*Vitis vinifera*, XM_002285033]
SSH24-7-C1	384	IN PROCESS	5×10^{-76}	1	Phosphate transporter, putative [*Vitis vinifera*, XM_002285117]
SSH24-1-G3	463	IN PROCESS	1×10^{-147}	6	D-3-phosphoglycerate dehydrogenase, putative [*Vitis vinifera*, XM_002285322]
SSH24-6-E1	559	IN PROCESS	1×10^{-54}	1	Serine/arginine rich splicing factor, putative [*Ricinus communis*, XM_002531442]
SSH24-6-H10	382	IN PROCESS	2×10^{-73}	2	Protein binding protein, putative [*Ricinus communis*, XM_002518657]
SSH24-11-H8	226	IN PROCESS	2×10^{-46}	1	Malate dehydrogenase, putative [*Populus trichocarpa*, XM_002298765]
SSH24-2-F8	722	IN PROCESS	1×10^{-155}	1	DnaJ-like protein [Populus trichocarpa, XM_002301212]
SSH24-10-G11	171	IN PROCESS	2×10^{-18}	1	UDP-glucose 4-epimerase [*Populus trichocarpa*, XM_002303617]
SSH24-8-G6	197	IN PROCESS	2×10^{-65}	1	Asparagine synthase, putative [*Populus trichocarpa*, XM_002306274]
SSH24-10-D10	379	IN PROCESS	3×10^{-49}	1	Syntaxin, putative [*Populus trichocarpa*, XM_002308290]
SSH24-3-C10	328	IN PROCESS	1×10^{-102}	2	Snf7, putative [*Populus trichocarpa*, XM_002310962]

Sequence ID [i]	Size (bp)	Accession n° [ii]	E-value	Redundancy (from 979 secuences)	Gene Identification
SSH24-6-H1	122	IN PROCESS	2×10^{-22}	1	60S ribosomal protein L13a protein [*Populus trichocarpa*, XM_002314563]
SSH24-5-C10	456	IN PROCESS	1×10^{-71}	1	Aldo-keto reductase, putative [*Populus trichocarpa*, XM_002321061]
SSH24-6-E3	240	IN PROCESS	6×10^{-28}	1	Zinc finger protein, putative [*Ricinus communis*, XM_002533198]
SSH24-6-F1	231	IN PROCESS	1×10^{-61}	1	Succinate dehydrogenase, putative [*Ricinus communis*, XM_002530436]
SSH24-5-C10	264	IN PROCESS	4×10^{-49}	1	Phosphatidic acid phosphatase, pustative [*Ricinus communis*, XM_002528028]
SSH24-2-F6	420	IN PROCESS	5×10^{-63}	1	Alpha/beta hydrolase domain containing protein 1,3, putative [*Ricinus communis*, XM_002526802]
SSH24-6-D2	313	IN PROCESS	5×10^{-43}	1	Cationic amino acid transporter, putative [*Ricinus communis*, XM_002526568]
SSH24-9-G10	303	IN PROCESS	2×10^{-28}	1	2OG-Fe(II) oxygenase, putative [*Ricinus communis*, XM_002524758]
SSH24-11-H7	507	IN PROCESS	2×10^{-89}	1	Cytochrome B5 isoform 1, putative [*Ricinus communis*, XM_002521050]
SSH24-5-A3	325	IN PROCESS	5×10^{-81}	1	Pyruvate dehydrogenase, putative [*Ricinus communis*, XM_002519618]
SSH24-10-H10	747	IN PROCESS	0.0	1	Transitional endoplasmic reticulum ATPase, putative
SSH24-3-E12	422	IN PROCESS	1×10^{-38}	2	Acyl-CoA thioesterase, putative [*Ricinus communis*, XM_002519297]
SSH24-2-B1	400	IN PROCESS	3×10^{-98}	2	60S ribosomal protein L13a protein [*Ricinus communis*, XM_002518061]
SSH24-3-E7	647	IN PROCESS	4×10^{-146}	1	60S ribosomal protein L7, putative [*Ricinus communis*, XM_002517470]
SSH24-1-E2	463	IN PROCESS	7×10^{-62}	1	Serine carboxypeptidase, putative [*Ricinus communis*, XM_002517243]
SSH24-8-G10	368	IN PROCESS	2×10^{-99}	2	Chaperone protein dnaJ, putative [*Ricinus communis*, XM_002514373]
SSH24-3-G4	858	IN PROCESS	0.0	2	Calnexin, putative [*Ricinus communis*, XM_002511020]
SSH24-7-B1	283	IN PROCESS	1×10^{-17}	1	Salutaridinol 7-O-acetyltransferase, putative [*Ricinus communis*, XM_002510620]

Sequence ID [i]	Size (bp)	Accession n° [ii]	E-value	Redundancy (from 979 secuences)	Gene Identification
SSH24-6-D6	142	IN PROCESS	2×10^{-18}	1	MAPEG-like protein [*Sorghum bicolor,* XM_002463967]
SSH24-5-H2	288	IN PROCESS	2×10^{-35}	1	RING-finger protein, putative [*Populus trichocarpa,* XM_002336479]
SSH24-2-E1	496	IN PROCESS	4×10^{-103}	1	Adenylyl-sulfate reductase, putative [*Populus trichocarpa,* XM_002332674]
SSH24-11-B2	328	IN PROCESS	3×10^{-102}	1	60S ribosomal protein L13a protein, putative [*Populus trichocarpa*, XM_002324016]
SSH24-7-B2	242	IN PROCESS	4×10^{-61}	4	Major pollen allergen Ory s 1 precursor, putative [*Populus trichocarpa,* XM_002326637]
SSH24-7-D1	371	IN PROCESS	5×10^{-82}	1	Protein translocase, putative [*Populus trichocarpa,* XM_002331008]
SSH24-3-C8	400	IN PROCESS	6×10^{-81}	3	Elongation factor 1 gamma, putative [*Populus trichocarpa,* XM_002325493]
SSH24-4-B3	175	IN PROCESS	5×10^{-14}	1	Transferase, putative [*Populus trichocarpa,* XM_002325418]
SSH24-6-F8	310	IN PROCESS	1×10^{-18}	1	NADP-dependent oxidoreductases, putative [*Vitis vinifera*, XM_002279323]
SSH24-6-A6	743	IN PROCESS	5×10^{-16}	3	Histone deacetylase [*Populus trichocarpa,* XM_002328042]
SSH24-8-H10	742	IN PROCESS	6×10^{-34}	1	Cytochrome P450 [*Vitis vinifera*, XM_002263573]
SSH24-2-H11	711	IN PROCESS	8×10^{-70}	1	Serine hydroxymethyltransferase 1 [*Glycine max,* EU912419]
SSH24-7-B10	511	IN PROCESS	2×10^{-26}	1	ADP ribosylating enzyme, putative [*Populus trichocarpa*, XM_002303403]
SSH24-7-H7	496	IN PROCESS	2×10^{-37}	1	Serine/Threonine protein kinases, putative [*Vitis vinifera*, XM_002274189]
SSH24-11-H3	491	IN PROCESS	6×10^{-13}	1	Oligonucleotide binding protein, putative [*Vitis vinifera*, XM_002282211]
SSH24-6-A5	388	IN PROCESS	5×10^{-12}	1	Lipase, putative [*Populus trichocarpa*, XM_002312776]
**Unidentified function genes**					
SSH24-6-B9	392	IN PROCESS	5×10^{-25}	2	Unknown [*Solanum lycopersicum*, AK247113]
SSH24-2-G1	125	IN PROCESS	4×10^{-19}	1	Unknown [*Solanum lycopersicum*, AK321416]
SSH24-6-A7	184	IN PROCESS	8×10^{-56}	1	Unknown [*Solanum lycopersicum*, AK323989]
SSH24-4-B4	253	IN PROCESS	4×10^{-49}	2	Unknown [*Oryza sativa*, CT830536]
SSH24-4-C6	479	IN PROCESS	2×10^{-57}	4	Unknown [*Vitis vinifera,* XM_002285205]

Sequence ID (i)	Size (bp)	Accession n° (ii)	E-value	Redundancy (from 979 secuences)	Gene Identification
SSH24-8-H3	215	IN PROCESS	2×10^{-53}	1	Unknown [*Populus trichocarpa*, XM_002315255]
SSH24-11-G7	338	IN PROCESS	3×10^{-79}	1	Unknown [*Populus trichocarpa*, XM_002324279]
SSH24-5-E9	330	IN PROCESS	8×10^{-105}	1	Unknown [*Daucus carota*, BI452005]
SSH24-3-C6	263	IN PROCESS	2×10^{-53}	2	Unknown [*Vitis vinifera*, XM_002264579]
**Novel genes**					
SSH24-2-A3	72	IN PROCESS		1	Unknown
SSH24-7-D3	93	IN PROCESS		1	Unknown
SSH24-2-F10	118	IN PROCESS		1	Unknown
SSH24-1-E7	122	IN PROCESS		1	Unknown
SSH24-2-A4	122	IN PROCESS		1	Unknown
SSH24-1-G8	133	IN PROCESS		1	Unknown
SSH24-8-H5	141	IN PROCESS		1	Unknown
SSH24-3-C12	150	IN PROCESS		1	Unknown
SSH24-2-C10	153	IN PROCESS		1	Unknown
SSH24-2-D2	164	IN PROCESS		2	Unknown
SSH24-2-B7	178	IN PROCESS		1	Unknown
SSH24-7-B7	179	IN PROCESS		1	Unknown
SSH24-4-G3	188	IN PROCESS		1	Unknown
SSH24-9-E11	191	IN PROCESS		1	Unknown
SSH24-5-A2	201	IN PROCESS		1	Unknown
SSH24-9-G6	210	IN PROCESS		1	Unknown
SSH24-4-A10	314	IN PROCESS		2	Unknown
SSH24-3-C3	218	IN PROCESS		1	Unknown
SSH24-10-G9	223	IN PROCESS		1	Unknown
SSH24-11-B6	234	IN PROCESS		2	Unknown
SSH24-2-H8	236	IN PROCESS		1	Unknown
SSH24-1-H2	253	IN PROCESS		1	Unknown
SSH24-7-E7	254	IN PROCESS		1	Unknown
SSH24-10-A12	254	IN PROCESS		1	Unknown
SSH24-5-C9	257	IN PROCESS		1	Unknown
SSH24-8-G1	265	IN PROCESS		1	Unknown
SSH24-5-A5	276	IN PROCESS		1	Unknown
SSH24-11-F7	276	IN PROCESS		1	Unknown
SSH24-4-G8	277	IN PROCESS		2	Unknown
SSH24-1-E10	279	IN PROCESS		1	Unknown
SSH24-8-G3	281	IN PROCESS		1	Unknown
SSH24-2-B11	282	IN PROCESS		1	Unknown
SSH24-10-B2	282	IN PROCESS		1	Unknown

Sequence ID (i)	Size (bp)	Accession n° (ii)	E-value	Redundancy (from 979 secuences)	Gene Identification
SSH24-9-D2	284	IN PROCESS		1	Unknown
SSH24-9-B7	286	IN PROCESS		1	Unknown
SSH24-3-B7	293	IN PROCESS		1	Unknown
SSH24-6-D11	314	IN PROCESS		1	Unknown
SSH24-6-C10	318	IN PROCESS		1	Unknown
SSH24-7-A5	320	IN PROCESS		1	Unknown
SSH24-9-E8	320	IN PROCESS		2	Unknown
SSH24-9-F2	316	IN PROCESS		1	Unknown
SSH24-6-G7	323	IN PROCESS		1	Unknown
SSH24-7-E5	323	IN PROCESS		1	Unknown
SSH24-7-F9	326	IN PROCESS		1	Unknown
SSH24-1-E3	327	IN PROCESS		1	Unknown
SSH24-7-A6	329	IN PROCESS		1	Unknown
SSH24-10-H11	329	IN PROCESS		1	Unknown
SSH24-6-B7	339	IN PROCESS		1	Unknown
SSH24-2-D5	368	IN PROCESS		1	Unknown
SSH24-4-C12	368	IN PROCESS		1	Unknown
SSH24-1-H4	371	IN PROCESS		1	Unknown
SSH24-11-G10	371	IN PROCESS		1	Unknown
SSH24-11-D5	375	IN PROCESS		1	Unknown
SSH24-4-H4	376	IN PROCESS		2	Unknown
SSH24-8-F7	380	IN PROCESS		1	Unknown
SSH24-9-E2	392	IN PROCESS		1	Unknown
SSH24-8-H1	394	IN PROCESS		1	Unknown
SSH24-3-B3	400	IN PROCESS		1	Unknown
SSH24-10-F1	404	IN PROCESS		1	Unknown
SSH24-6-A8	410	IN PROCESS		1	Unknown
SSH24-9-E12	415	IN PROCESS		1	Unknown
SSH24-8-B5	418	IN PROCESS		1	Unknown
SSH24-9-A7	421	IN PROCESS		1	Unknown
SSH24-3-E9	424	IN PROCESS		4	Unknown
SSH24-11-D12	443	IN PROCESS		1	Unknown
SSH24-5-A11	460	IN PROCESS		1	Unknown
SSH24-11-A7	460	IN PROCESS		1	Unknown
SSH24-6-F5	462	IN PROCESS		1	Unknown
SSH24-7-E1	462	IN PROCESS		2	Unknown
SSH24-9-G7	483	IN PROCESS		1	Unknown
SSH24-11-E8	483	IN PROCESS		1	Unknown

Sequence ID (i)	Size (bp)	Accession n° (ii)	E-value	Redundancy (from 979 secuences)	Gene Identification
SSH24-1-G4	505	IN PROCESS		1	Unknown
SSH24-7-H1	513	IN PROCESS		1	Unknown
SSH24-10-F3	517	IN PROCESS		2	Unknown
SSH24-10-C12	522	IN PROCESS		1	Unknown
SSH24-8-F11	567	IN PROCESS		1	Unknown
SSH24-11-E9	584	IN PROCESS		1	Unknown
SSH24-3-H1	823	IN PROCESS		1	Unknown
SSH24-2-A9	798	IN PROCESS		1	Unknown
SSH24-10-D8	139	IN PROCESS		1	Unknown
SSH24-3-E6	302	IN PROCESS		1	Unknown
SSH24-3-A3	309	IN PROCESS		1	Unknown
SSH24-7-G12	314	IN PROCESS		1	Unknown
SSH24-2-F12	339	IN PROCESS		1	Unknown
SSH24-1-G9	354	IN PROCESS		12	Unknown
SSH24-6-E2	511	IN PROCESS		1	Unknown
SSH24-7-B3	511	IN PROCESS		1	Unknown
SSH24-7-G2	453	IN PROCESS		1	Unknown
SSH24-8-B10	433	IN PROCESS		1	Unknown
SSH24-9-D11	408	IN PROCESS		1	Unknown
SSH24-7-A4	358	IN PROCESS		1	Unknown
SSH24-10-C4	358	IN PROCESS		1	Unknown
SSH24-2-G5	365	IN PROCESS		2	Unknown
SSH24-5-B1	375	IN PROCESS		1	Unknown

(i) Identification number on the subtractive library.

Table S6. Sequences of primers used for real-time quantitative reverse transcription PCR (qRT-PCR) analyses.

Putative gene identification	Sequence for forward (F) and reverse (R) primers (5'→3')
Signaling molecules biosynthesis genes	
S-adenosyl-L-methionine synthetase 1 [*Daucus carota*, AY583461]	F: GCAACAACAGGTTCCTGAAGAC<br>R: TCCCACTTGAGGGGCTTGACTA
1-aminocyclopropane-1-carboxylate synthase [*Carica papaya*, AM113989]	F: GCTGTTTCTCGCTAATTTGCTG<br>R: ACTGGAAGAAATCGGCATCAAG
Lipoxygenase 2 [*Actinidia deliciosa*, DQ497797]	F: TTAAGAGGGGAATGGCAGTTGAG<br>R: GTCTTGTAGTAGAAGCCGCAGTA
Lipoxygenase 5 [*Actinidia deliciosa*, DQ497796]	F: CGACCCTGATGGCGTTTACCT<br>R: TCCAATCTGGGATGTCTCGTTGT
12-oxophytodienoate reductase, putative [*Vitis vinifera*, XM_002281083]	F: GGATGAAAAGTGTAGGAGAGAAG<br>R: CGTAACCCCCAGCAACAATAAA
Omega-6 fatty acid desaturase [*Petroselinum crispum*, U86072]	F: ACAACTCATCGGAATGGGATTGG<br>R: AGTATCAGTGATGTTATGGAGCACC
3-ketoacyl-CoA thiolase B [*Mangifera indica*, X75329]	F: CATTTAAGAAGGATGGGACCACA<br>R: GCGGCAAAACTCCTGAATACACC
NADH-ubiquinone oxidoreductase, putative [*Prunus armeniaca*, AF139496]	F: GTGCCAGAAACACTGCCAGAG<br>R: AAGATCAATCCATCCGCCTCAG
Glycolate oxidase, putative [*Vitis vinifera*, XM_002278068]	F: TGATGGTGAGGCTGGTGTGAGT<br>R: GTGTGATCTCTTTGATTGAGCGG
NADPH oxidase, putative [*Solanum tuberosum*, AB198716]	F: TGGCATCCTTTTTCTATCACTTCT<br>R: CACTTAGATAATCATCATCTGGG
MnSOD, putative [*Pisum sativum,* X60170]	F: CAGTTTGGTACATTGGAGGCG<br>R: GGGTCCTGATTTGCAGTGGTCT
Primary metabolism genes	
3-deoxy-D-arabino-heptulosonate [*Petroselinum crispum*, AF012864]	F: GATGCTATCAGGGCTGAAGTCAA<br>R: CCTAGATCATCAAAAGTCACGGTTC
5-enolpyruvylshikimate 3-phosphate synthase [*Camptotheca acuminata*, AY639815]	F: TAGGAGCAACAGTTGAAGAGGGT<br>R: ATCTGAACAGGCAGCAAGAGAGA

Table S6. continued.

Putative gene identification	Sequence for forward (F) and reverse (R) primers (5'→3')
Secondary metabolism genes	
4-coumarate-coa ligase [*Populus trichocarpa*, XM_002304328]	F: GGGACTACTGGGACTTCCAAAG<br>R: AGCCAAATAAACCATCTCCCAGC
Phenylalanine ammonia-lyase (gDcPAL1) [*Daucus carota*, D85850]	F: AGGGCAAACCCGAATTTACTGAC<br>R: CATAAGAGCTTCCATCCAAGATG
Trans-cinnamate 4-monooxygenase [*Petroselinum crispum*, L38898]	F: CCTGGAATCATCCTTGCATTGC<br>R: TGTCCTCCTTTCTCTGCTGTGT
Chalcone synthase 2 [*Daucus carota*, AJ006780]	F: CCACTATTCGGCTCCCTCGTT<br>R: CTCTGATTTGGAGTGTCCCCA
Caffeoyl-CoA 3-O-methyltransferase [*Petroselinum crispum*, M69184]	F: TATTCTCTCCTTGCCACTGCTCT<br>R: CCCTCTCTGAAGTCAATTTTGTG
Cinnamyl alcohol dehydrogenase [*Aralia cordata*, D13991]	F: GGAGGCAATGGATCATCTTGGT<br>R: GAGAGGTAAGGTTCGAGTGGGT
Bergaptol O-methyltransferase [*Glehnia littoralis*, AB363638]	F: CTATGGTTCAACGCATCCTCC<br>R: TGTCCAGCAATAGAGCCACTC
